# The RNA-binding protein QKI governs a muscle-specific alternative splicing program that shapes the contractile function of cardiomyocytes

**DOI:** 10.1093/cvr/cvad007

**Published:** 2023-01-11

**Authors:** Pablo Montañés-Agudo, Simona Aufiero, Eva N Schepers, Ingeborg van der Made, Lucia Cócera-Ortega, Auriane C Ernault, Stéphane Richard, Diederik W D Kuster, Vincent M Christoffels, Yigal M Pinto, Esther E Creemers

**Affiliations:** Department of Experimental Cardiology, Amsterdam UMC, University of Amsterdam, Meibergdreef 15, 1105AZ Amsterdam, The Netherlands; Department of Experimental Cardiology, Amsterdam UMC, University of Amsterdam, Meibergdreef 15, 1105AZ Amsterdam, The Netherlands; Department of Clinical Epidemiology, Biostatistics and Bioinformatics, Amsterdam UMC, University of Amsterdam, Meibergdreef 9, Amsterdam, The Netherlands; Department of Experimental Cardiology, Amsterdam UMC, University of Amsterdam, Meibergdreef 15, 1105AZ Amsterdam, The Netherlands; Department of Experimental Cardiology, Amsterdam UMC, University of Amsterdam, Meibergdreef 15, 1105AZ Amsterdam, The Netherlands; Department of Experimental Cardiology, Amsterdam UMC, University of Amsterdam, Meibergdreef 15, 1105AZ Amsterdam, The Netherlands; Department of Experimental Cardiology, Amsterdam UMC, University of Amsterdam, Meibergdreef 15, 1105AZ Amsterdam, The Netherlands; Segal Cancer Center, Lady Davis Institute for Medical Research and Gerald Bronfman Department of Oncology and Departments of Biochemistry, Human Genetics and Medicine, McGill University, Montréal, QC, Canada H3T 1E2; Department of Medical Physiology, Amsterdam UMC, Vrije Universiteit Amsterdam, De Boelelaan 1117, Amsterdam, Netherlands; Department of Medical Biology, Amsterdam UMC, University of Amsterdam, Meibergdreef 9, Amsterdam, The Netherlands; Department of Experimental Cardiology, Amsterdam UMC, University of Amsterdam, Meibergdreef 15, 1105AZ Amsterdam, The Netherlands; Department of Experimental Cardiology, Amsterdam UMC, University of Amsterdam, Meibergdreef 15, 1105AZ Amsterdam, The Netherlands

**Keywords:** Alternative splicing, RNA-binding proteins, Quaking, Cardiomyocytes

## Abstract

**Aims:**

In the heart, splicing factors orchestrate the functional properties of cardiomyocytes by regulating the alternative splicing of multiple genes. Work in embryonic stem cells has shown that the splicing factor Quaking (QKI) regulates alternative splicing during cardiomyocyte differentiation. However, the relevance and function of QKI in adult cardiomyocytes remains unknown. In this study, we aim to identify the *in vivo* function of QKI in the adult mouse heart.

**Methods and results:**

We generated mice with conditional deletion of QKI in cardiomyocytes by the Cre-Lox system. Mice with cardiomyocyte-specific deletion of QKI died during the foetal period (E14.5), without obvious anatomical abnormalities of the heart. Adult mice with tamoxifen-inducible QKI deletion rapidly developed heart failure associated with severe disruption of sarcomeres, already 7 days after knocking out QKI. RNA sequencing revealed that QKI regulates the alternative splicing of more than 1000 genes, including sarcomere and cytoskeletal components, calcium-handling genes, and (post-)transcriptional regulators. Many of these splicing changes corresponded to the loss of muscle-specific isoforms in the heart. Forced overexpression of QKI in cultured neonatal rat ventricular myocytes directed these splicing events in the opposite direction and enhanced contractility of cardiomyocytes.

**Conclusion:**

Altogether, our findings show that QKI is an important regulator of the muscle-specific alternative splicing program that builds the contractile apparatus of cardiomyocytes.

## Graphical Abstract

**Graphical Abstract:**
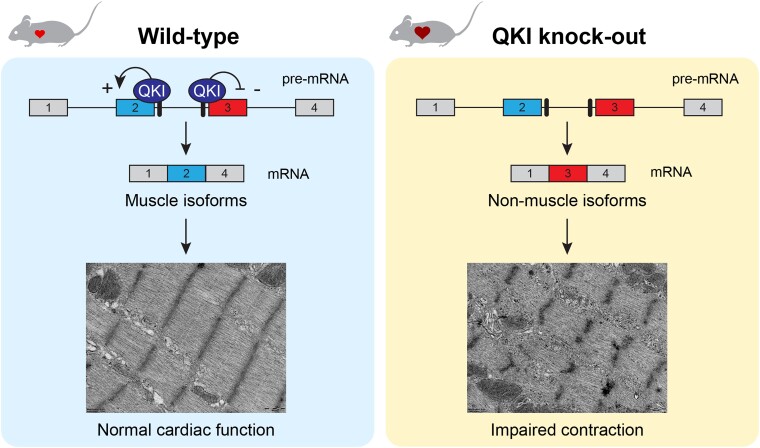

## 1. Introduction

Alternative splicing, the process by which exons from one mRNA can be spliced in different arrangements, is a crucial mechanism to expand protein diversity. It is now established that more than 95% of all human genes undergo alternative splicing to produce protein isoforms that are functionally or structurally specialized for the cell type in which they are expressed.^1^ In the heart, for example, the contractile properties of the sarcomere are precisely orchestrated through alternative splicing of titin, troponin T, and tropomyosin to meet the varying demands of the heart during development and disease.^1,2^ One mechanism to precisely regulate alternative splicing in a tissue- and developmental stage-specific manner is via the expression of splicing factors. RBM20 is the most studied splicing factor in the heart, which has been shown to regulate networks of splicing events, particularly in sarcomere and calcium-handling genes.^3–5^ Mutations in *RBM20* are a frequent cause of familial dilated cardiomyopathy (DCM) which highlights the functional impact of aberrant splicing in the heart.^6,7^ Loss-of-function studies in mice also uncovered roles for other muscle restricted-splicing factors, namely RBM24 and RBFOX1 in the heart,^8,9^ but the list of RNA-binding proteins (RBPs) controlling alternative splicing in the heart is far from complete.

The splicing factor Quaking (QKI) is a member of the Signal Transduction and Activator RNA (STAR) family of RBPs, which is ubiquitously expressed with highest levels in brain and heart.^10^ QKI has three main isoforms, i.e. QKI-5, QKI-6, and QKI-7, that differ in the C-terminal region. QKI-5 is the predominant isoform in the heart and is the only one with a nuclear localization signal, which allows it to act as a splicing factor by binding nuclear pre-mRNAs.^11^ QKI-6 and QKI-7 are cytosolic proteins, and they are mainly involved in binding mRNAs outside of the nucleus to control their stability.^11,12^ The role of QKI in the nervous system has been well studied, in fact, the name ‘quaking’ stems from the phenotype of the ‘quaking viable’ mouse model that has reduced levels of myelin in its nervous system, leading to a shaking behaviour, with frequent seizures.^13–15^ In the heart, the function of QKI is not fully understood. Its activity in cardiomyocytes has been associated with a better outcome after ischaemia/reperfusion (I/R)^16,17^ and after doxorubicin toxicity.^18^ Systemic loss of QKI in mice (using the *Qki^betaGeo^* mouse line) resulted in embryonic lethality around Day E9.5–E10.5, which was attributed to the failure of blood circulation in the yolk sac, in which vascular remodelling was impaired.^19^ Other abnormalities that were observed at this embryonic stage were open neural tubes, incomplete embryonic turning, and pericardial effusion, while the hearts seemed normal.^19^ In a subsequent study using the same *Qki^betaGeo^* mouse line, several splicing changes were detected in the hearts of the KO embryos.^20^ Work in human embryonic stem cell-derived cardiomyocytes (hESC-CM) indicated that QKI is required for the proper splicing of genes involved in myofibrillogenesis, such as *ACTN2*, *NEBL*, and *TTN*.^20^ Despite this first evidence for splicing regulation during differentiation of cardiac progenitors to early cardiomyocytes and in the specification of cardiac mesoderm,^21^ the functional relevance of QKI-mediated splicing in the embryonic and adult heart remains unknown.

In this study, we investigated the function of QKI in the mouse heart by selectively deleting QKI in cardiomyocytes. To circumvent embryonic lethality due to impaired yolk sac development and to be able to ascertain a cardiomyocyte-autonomous role for QKI, we utilized the Cre-LoxP system to conditionally delete QKI from cardiomyocytes. Mice with cardiac-specific deletion of QKI died during the early foetal stages (E14.5), without obvious structural abnormalities of the heart. In the adult myocardium, loss of QKI (using the tamoxifen-inducible MerCreMer) induced dilation of the ventricles and a rapid decline in cardiac function, which was associated with severe disruption of sarcomere organization. RNA sequencing revealed splicing changes in more than a thousand genes in the adult QKI KO hearts, a large subset of which represents muscle-specific isoforms. While deletion of QKI in adult myocardium induced a rapid heart failure phenotype, we also show that forced overexpression of QKI in neonatal cardiomyocytes is able to enhance sarcomere contractility. Altogether, we are the first to show that QKI is a crucial splicing factor in the adult heart to maintain a muscle-specific splicing program.

## 2. Methods

### 2.1 Animal studies

Animal studies were approved by the Institutional Animal Care and Use Committee of the University of Amsterdam and carried out in compliance with the guidelines of this institution and the Directive 2010/63/EU of the European Parliament. Animal husbandry was performed by the Animal Research Institute AMC.

Cardiomyocyte-specific knock-out mice of *Qki* were generated by crossing the previously generated *Qki*-floxed mice^13^ with the conditional *Myh6-Cre* (Jackson Laboratory stock #011038) and with the tamoxifen-inducible *Myh6-MerCreMer* (Jackson Laboratory stock #005657)^22^ in C57BL/6N background. In all experiments, mice had a single copy of the *Cre* or the *MerCreMer* (MCM) recombinase allele. Males and females were included in the experiments.

For inducing *MerCreMer* recombinase activity, a tamoxifen solution (2.5 mg/mL tamoxifen in 10% ethanol, 90% sunflower oil) was injected intraperitoneally for 4 consecutive days (total dose: 100 mg tamoxifen/kg mouse) in 12–17-week-old mice (*n* per group: 5 ciQKI WT, 6 ciQKI HET, and 9 ciQKI KO).

Echocardiography and electrocardiogram (ECG) recordings were performed as indicated in the Supplemental Methods.

### 2.2 RNA and protein analysis

For adult mice, total RNA was extracted from the left ventricle by using TRI reagent (Sigma, Ref T9424) following the manufacturer’s instructions. For E12.5 embryos, RNA from both ventricles was extracted using the kit ReliaPrep RNA Miniprep Systems (Promega, Ref Z6111). RNA quality was determined using the Agilent RNA 6000 Nano Kit and the Agilent 2100 Bioanalyser. All samples had an RNA Integrity score ≥8. cDNA synthesis and (q)RT–PCRs were performed as described in the Supplemental Methods. Primer sequences are found in Supplementary material online, *Table S1*.

Western blotting and histology were performed as depicted in the Supplemental Methods. Antibodies and their dilutions are listed in Supplementary material online, *Table S2*.

### 2.3 RNA sequencing

RNA from left ventricle samples of five adult ciQKI WT and five adult ciQKI KO mice, and four cQKI WT and four cQKI KO E12.5 ventricles was used for paired-end RNA sequencing. Details of library preparation, RNA sequencing, and data analysis are found in the Supplemental Methods.

### 2.4 Differential gene expression and exon usage

Differential gene expression analysis was performed using the R Bioconductor package, DESeq2^23^ (Bioconductor release 3.13). Transcripts Per Million (TPM) for each gene was also calculated. Genes with TPM value ≥0.5, an absolute log_2_ fold change cut-off ≥1.0, and adjusted *P*-value cut-off ≤ 0.05 were deemed significantly differentially expressed.

Differential exon usage analysis was performed using the R Bioconductor package, DEXSeq^24^ (Bioconductor release 3.13). Only genes with a TPM value ≥ 0.5 were considered. Exons with an absolute log_2_ fold change cut-off ≥ 1.0, and adjusted *P*-value cut-off ≤ 0.05 were deemed significantly differentially spliced.

### 2.5 Neonatal rat ventricular myocytes

Isolation, transduction, culture and contraction measurements of neonatal rat ventricular myocytes (NRVMs) are depicted in the Supplemental Methods.

### 2.6 Statistics

Data were analysed using GraphPad Prism 9 (GraphPad, San Diego, CA, USA). Data are presented as mean ± standard deviation unless differently stated. Results were analysed with appropriate statistical tests, as indicated in the respective figure legends. A value of *P* < 0.05 was considered statistically significant.

## 3. Results

### 3.1 Cardiomyocyte-specific removal of QKI causes embryonic lethality

**Figure 1:**
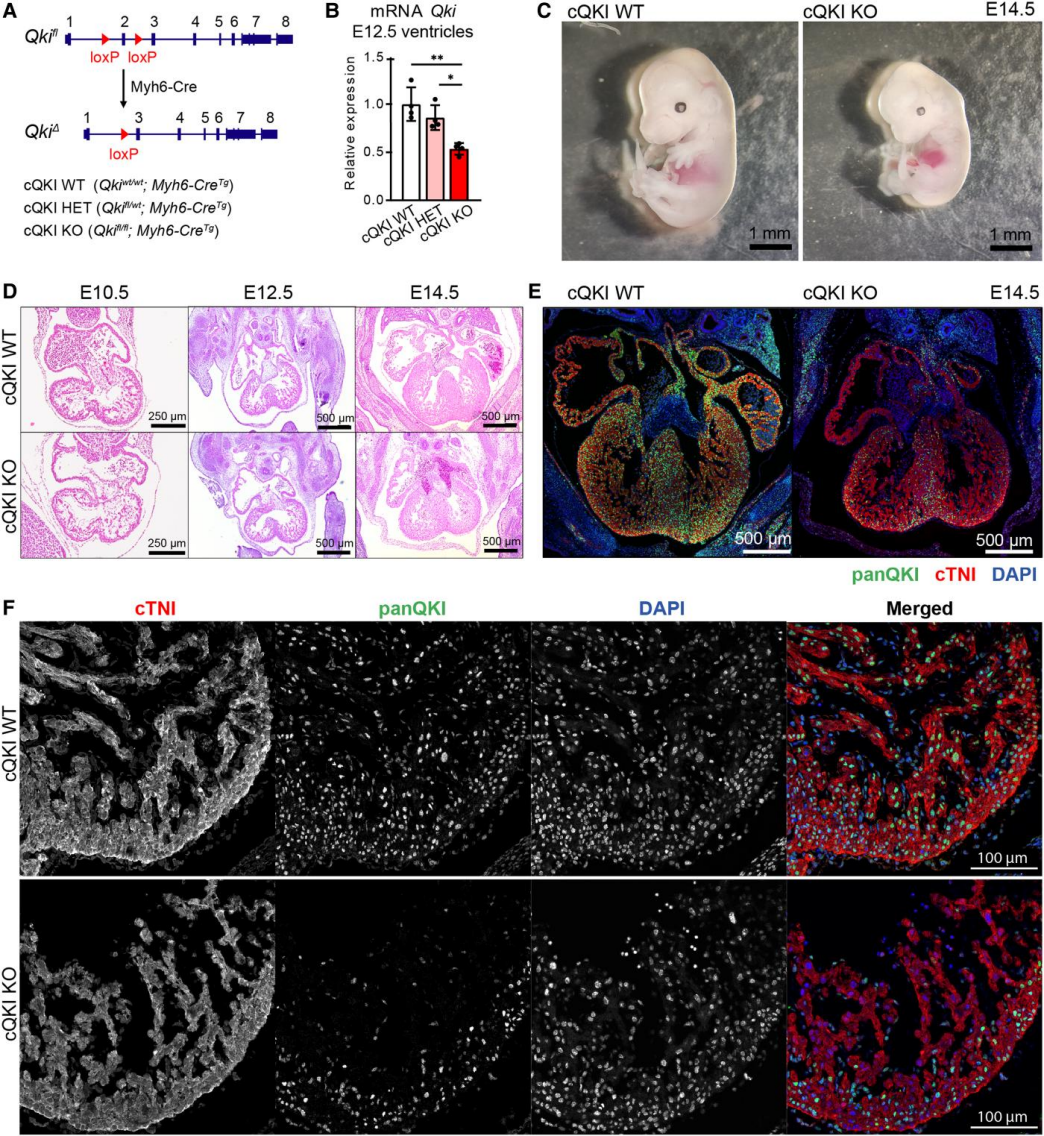
QKI deletion in cardiomyocytes results in early embryo lethality. (*A*) Schematic drawing of the floxed *Qki* allele. (*B*) *Qki* mRNA levels in ventricles at E12.5. *n* = 4 embryos per group. One-way analysis of variance (ANOVA) followed by Tukey’s multiple comparison test; **adjusted *P* < 0.01; *adjusted *P* < 0.05. (*C*) Representative images of mouse embryos at E14.5. Scale bar: 1 mm. (*D*) H&E stainings depicting a four-chamber view of embryonic hearts at indicated developmental timepoints. Scale bar: 250 µm E10.5, 500 µm E12.5–14.5. (*E*) Four-chamber view of E14.5 hearts and (*F*) magnification of the LV at E12.5 immunocytochemically stained with DAPI (blue), anti-panQKI (green), and anti-cTNI (red) antibodies. Scale bar: 500 µm E14.5, 100 µm E12.5. Representative images of three embryos per group and per timepoint are shown.

To investigate the function of the splicing factor QKI in the heart, we generated a cardiomyocyte-specific QKI knockout mouse model by crossing *Qki*-floxed mice^13^ with mice carrying the *Myh6-Cre* transgene,^25^ in which the onset of Cre expression is as early as embryonic stage E7.5.^26^ In this conditional knockout allele, exon 2 of *Qki* is flanked by loxP sites. Exon 2 encodes part of the KH RNA-binding domain, common to all QKI isoforms, and its deletion generates a frameshift, resulting in a null allele^13^ (*Figure 1A*). We refer to the homozygous *Qki^fl/fl^*; *Myh6-Cre^Tg^* as conditional QKI knock-out mice (cQKI KO), *QKI^fl/wt^*; *Myh6-Cre^Tg^* as conditional QKI heterozygous mice (cQKI HET), and *Qki^wt/wt^*; *Myh6-Cre^Tg^* as conditional QKI wild-type (cQKI WT) mice.

cQKI HET mice were born at the expected Mendelian ratios and were viable, but cQKI KO mice were not found at birth, indicating that QKI expression in cardiomyocytes is essential during embryonic development (Supplementary material online, *Table S3*). To pinpoint the timepoint of embryonic death, we collected embryos at different stages and found cQKI KO embryos at expected Mendelian ratios until Day E14.5 (Supplementary material online, *Table S4*). From that timepoint onwards, all cQKI KO embryos were dead or reabsorbed. At E14.5, cQKI KO embryos displayed pericardial effusion and signs of necrosis (*Figure 1C*). No differences in embryo and heart size were detected between the three genotypes at E10.5 and E12.5 (Supplementary material online, *Figure S1*). Furthermore, Hematoxylin & Eosin (H&E) stainings did not reveal obvious defects in cardiac anatomy at Day E10.5, E12.5, or E14.5 (*Figure 1D*), suggesting the QKI cKO hearts were functionally compromised, not anatomically.

To assess the deletion efficiency by Myh6-Cre, we performed qRT–PCR on RNA isolated from E12.5 ventricles using primers spanning exons 2 and 3 of *Qki*. This showed deletion levels of ∼15% in cQKI HET and ∼50% in cQKI KO hearts (*Figure 1B*). Of note, QKI is also expressed at lower levels in other cell types of the heart (e.g. vascular smooth muscle cells, fibroblasts, and macrophages), which may contribute to the background expression of QKI in the knockout hearts. Next, we validated the knock-out approach by immunostainings for QKI and the cardiomyocyte marker cardiac troponin I (cTNI). In WT embryos, we could detect QKI protein in cardiomyocytes of the four chambers of the developing heart, however, QKI expression was clearly reduced in the QKI cKO embryos at Days E12.5 and E14.5 (*Figure1E* and *F*, Supplementary material online, *Figure S2*). At E14.5, residual QKI expression was observed particularly in cells of the compact myocardium and the middle axis of the heart. We do not have a clear explanation for the pattern of remaining cells, but it could be explained by cardiomyocytes not expressing the Cre recombinase or by other cell types in that area expressing QKI.

In conclusion, the heart-specific cQKI KO mice were unable to progress beyond embryonic day E14.5. This is substantially later than the lethality caused by whole-body ablation of QKI, which occurs around E9.5, and which has been attributed to abnormalities in vascular remodelling of the yolk sac.^27^ Overall, this indicates that QKI regulates key processes in cardiomyocytes during development.

### 3.2 Cardiac-restricted removal of QKI at adulthood causes heart failure

**Figure 2:**
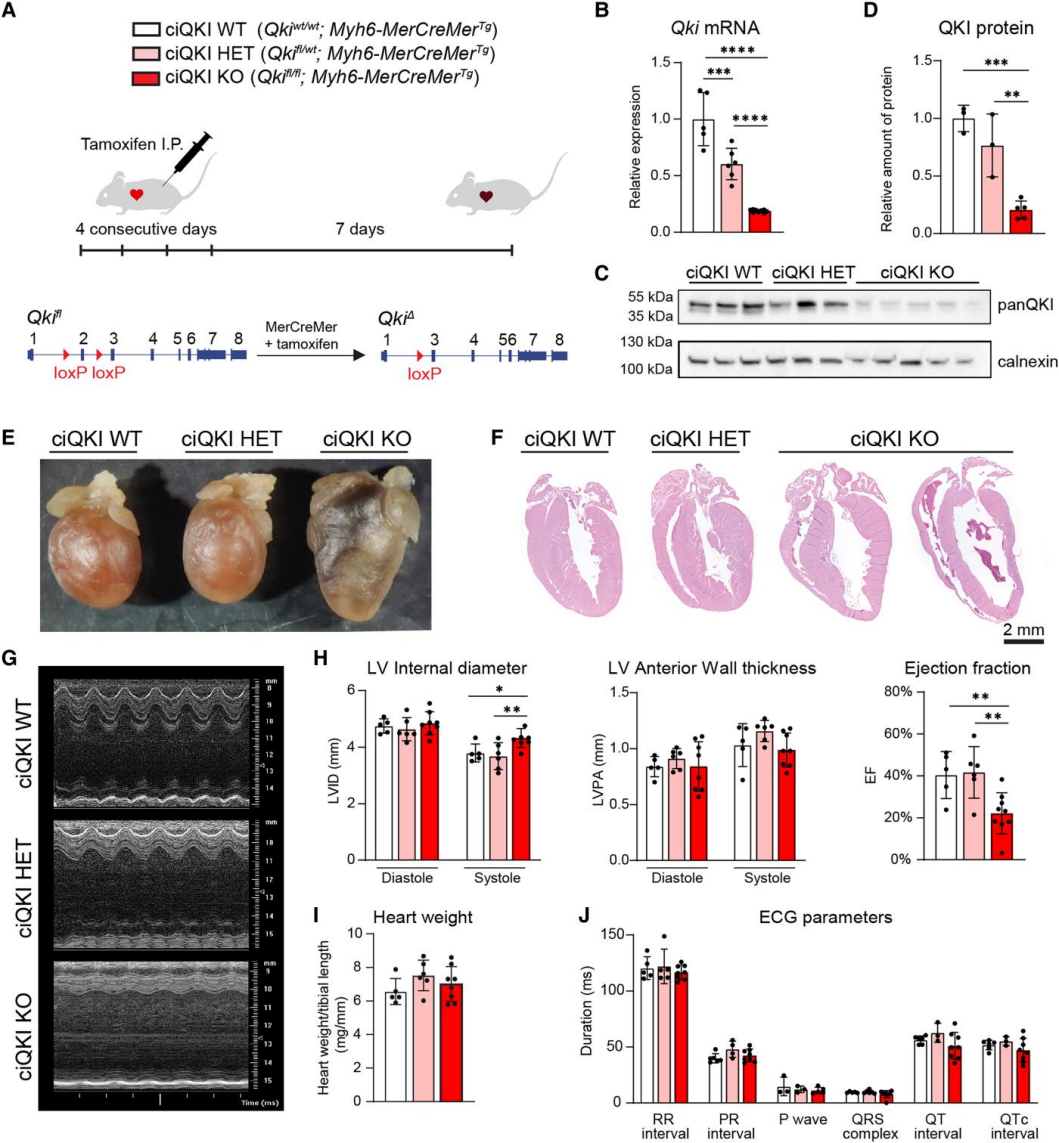
Deletion of QKI in adult cardiomyocytes induces heart failure. (*A*) Experimental approach to knock out QKI in adult cardiomyocytes by the Cre/Lox system. (*B*) *Qki* mRNA levels in LV tissue as measured by qPCR. (*C*) QKI protein levels in LV as measured by western blot and (*D*) its quantification. (*E*) Representative pictures of hearts after paraformaldehyde fixation. (*F*) H&E stainings depicting WT, HET, and KO hearts. Scale bar: 2 mm. (*G*) Representative echocardiographic M-mode traces. (*H*) LV internal diameter, LV anterior wall thickness, and EF, measured during diastole and systole using echocardiography. (*I*) Normalized heart weight. (*J*) Electrocardiogram parameters. All data and images correspond to the same experiment, which ended 7 days after the last tamoxifen injection. Number of mice per group: five ciQKI WT, six ciQKI HET, and nine ciQKI KO. Data are mean ± standard deviation. One-way ANOVA followed by uncorrected multiple comparison Fisher’s least significant difference; ****P < 0.0001; ***P < 0.001; **P < 0.01; *P < 0.05.

To overcome embryonic lethality and to be able to study QKI function in the adult heart, we created a cardiac-specific tamoxifen-inducible QKI (ciQKI) knockout mouse line by crossing the *Qki*-floxed mice with mice carrying the *Myh6-mER-Cre-mER* transgene (*Myh6-MCM^Tg^*).^22^ To induce the Cre-loxP recombination, we injected tamoxifen intraperitoneally in 12- to 17-week-old WT (*Qki^wt/wt^*, *Myh6-MCM^tg^*), ciQKI HET (*Qki^fl/wt^*, *Myh6-MCM^tg^*), and ciQKI KO (*Qki^fl/fl^*, *Myh6-MCM^tg^*) mice for 4 consecutive days (*Figure 2A*). We noted that within 5 days after the last tamoxifen injection a subset of ciQKI KO mice started to become inactive, while the tamoxifen-injected WT and ciQKI HET mice appeared healthy. As low physical activity can be indicative of heart failure in the ciQKI KO group, we terminated the experiment 7 days after the last tamoxifen injection. qRT–PCR on heart tissue revealed that *Qki* mRNA levels were reduced by 80% in the ciQKI KO and by 40% in the ciQKI HET hearts, 7 days after the last tamoxifen injection (*Figure 2B*). QKI protein levels were reduced by 80% in ciQKI KO mice, as shown by western blotting (*Figure2C* and *D*).

Inspection of global cardiac morphology and histological analysis on H&E-stained sections, 7 days after the last tamoxifen injection, showed enlargement and thinning of the ventricles (*Figure2E* and *F*). Echocardiography confirmed a heart failure phenotype with severe systolic dysfunction in ciQKI KO mice. Specifically, ejection fraction (EF) was reduced by ∼45% in ciQKI KO mice compared with the cQKI WT, and this was associated with a ∼14% increase in left ventricular (LV) internal diameter during systole (*Figure2G* and *H*, Supplementary material online, *Table S5*). No differences in heart weight and ECG parameters were found in ciQKI KO mice (*Figure2I* and *J*, Supplementary material online, *Figure S3*, *Table S5*).

**Figure 3:**
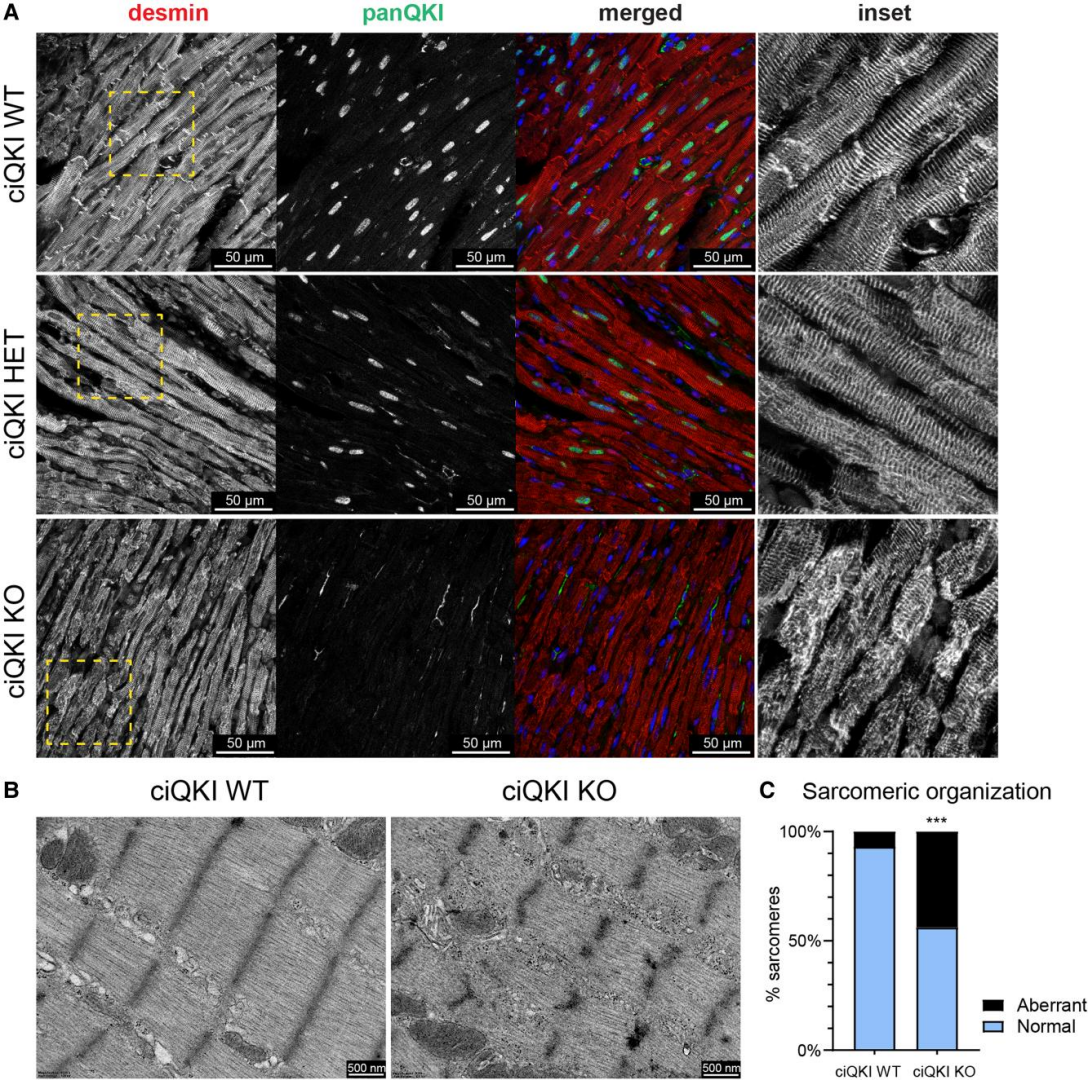
Loss of QKI disrupts cytoskeletal and sarcomere organization in the adult heart. (*A*) Immunocytochemistry showing the cytoskeletal protein desmin (red), panQKI (green), and DAPI (blue). The right panels (inset) represent magnifications of the yellow box in the desmin panels. Scale bar: 50 µm. (*B*) Representative transmission electron microscopy images of cardiomyocytes from ciQKI WT and ciQKI KO mice. Scale bar: 500 nm. (*C*) Quantification of aberrant sarcomeres in LV cardiomyocytes in electron microscopy images. Three mice per group and four images per mouse. *χ*^2^ test. ***adjusted *P* < 0.001.

Next, we performed double immunocytochemistry for QKI and desmin (an intermediate filament connecting sarcomeric Z-discs to the cytoskeleton) on adult ciQKI WT, ciQKI HET, and ciQKI KO hearts (*Figure 3A*) and evaluated them by confocal microscopy. This showed, besides a nearly complete loss of QKI protein, that the organization of the myofibrils was disrupted in the ciQKI KO hearts. Immunohistochemistry for alpha-actinin (ACTN2, which anchors actin filaments to the Z-disc) and titin (TTN, a molecular spring that connects the Z-line to the M-line in the sarcomere) yielded a comparable picture of disorganized myofilaments (Supplementary material online, *Figure S4* and *S5*). Of note, in the ciQKI KO hearts, we observed variation in the extent of filaments disruption, as some regions were more affected than others. No disrupted filaments were observed in the WT or ciQKI HET hearts. Ultrastructural analysis of the adult myocardium using transmission electron microscopy showed these defects in greater detail (*Figure3B* and *C*). Whereas WT hearts exhibited intact sarcomeres with clearly defined Z-discs and well-organized myofibrils, ciQKI KO hearts displayed degenerated sarcomeres, and this was associated with glycogen deposits around sarcomeres (Supplementary material online, *Figure S6*).

In conclusion, we show that QKI depletion in the adult myocardium leads to a DCM that rapidly progresses into heart failure and provide evidence that disrupted sarcomeric organization underlies this contractile defect.

### 3.3 QKI is required for alternative splicing in cardiomyocytes

To elucidate the mechanism underlying the observed functional and ultrastructural defects, we performed RNA sequencing in adult (five WT and five ciQKI KO hearts, 7 days after tamoxifen injections) and in E12.5 embryonic hearts (four WT and four cQKI KO). To be able to analyse gene expression and splice isoforms, cDNA libraries were subjected to ultra-deep sequencing, with around 250 million paired-end reads per sample.

**Figure 4:**
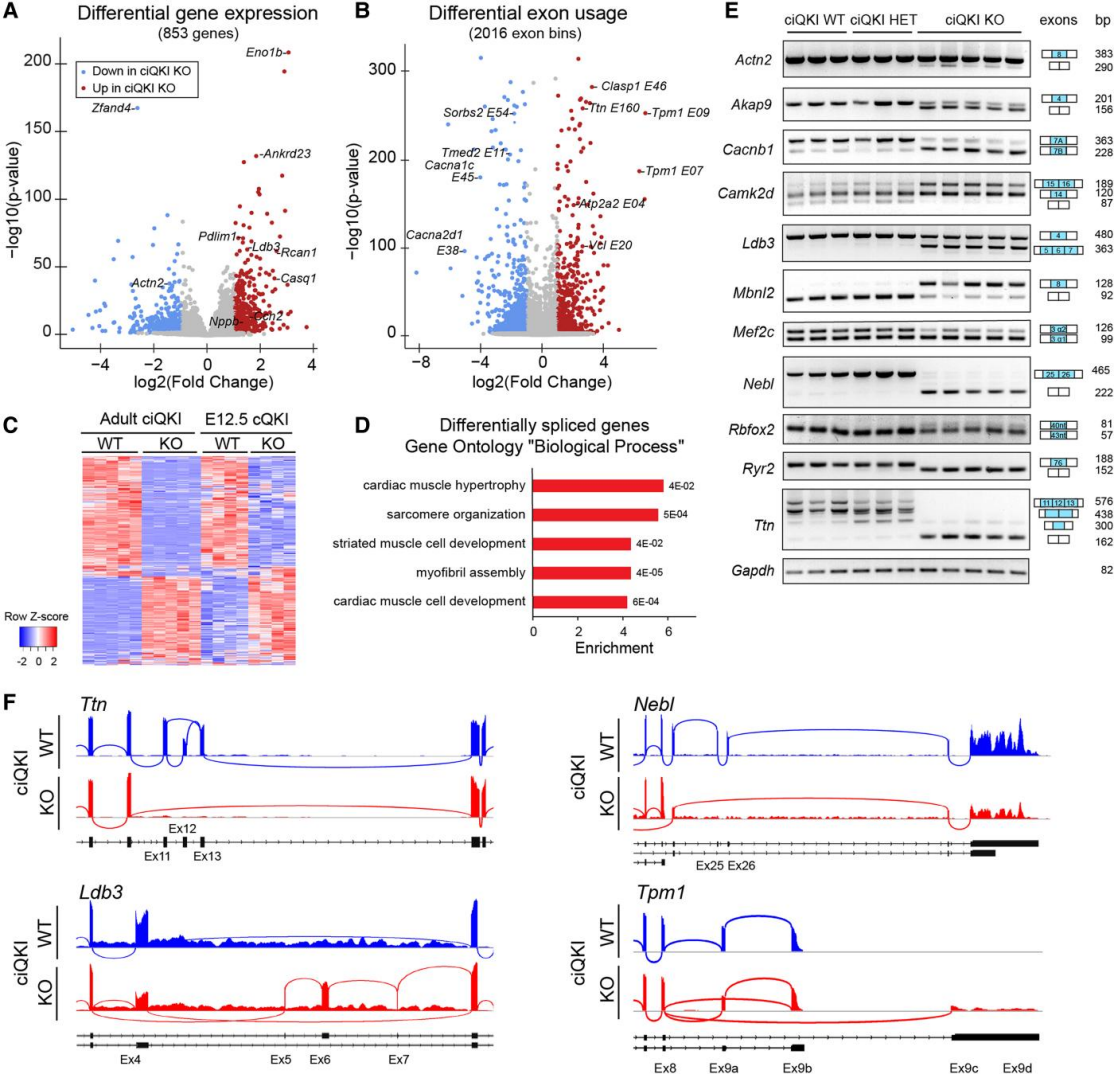
QKI is required for alternative splicing in cardiomyocytes of the adult heart. (*A*) Volcano plots showing differential gene expression and (*B*) differential exon usage of five ciQKI WT and five ciQKI KO hearts, 7 days after tamoxifen injections. (*C*) Heatmap showing differential exon usage after QKI removal in adult LV (*n* = 5 mice per group) and in embryonic ventricles (*n* = 4 mice per group). *Z*-scores were calculated in adults and embryos separately to illustrate the overlap in QKI targets between embryos and adults. (*D*) Pathway analysis of differentially spliced genes in the adult hearts for ‘biological process’ GO terms. (*E*) Validation of QKI-mediated splicing events by RT–PCR in three ciQKI WT, three ciQKI HET, and five ciQKI KO hearts (adults). (*F*) Representative sashimi plots of ciQKI WT and ciQKI KO hearts (adult), illustrating detailed splicing changes in *Ttn*, *Nebl*, *Ldb3*, and *Tpm1*. Exon numbers and transcript isoforms are indicated below the plot.

We first performed differential gene expression analysis on the adult hearts, and we found 409 genes significantly up-regulated and 444 down-regulated in the ciQKI KO hearts (*Figure 4A*, Online data 1). Among the up-regulated genes, there were markers of cardiac stress, such as *Acta1*, *Casq2*, *Ccn2*, *Myh7*, *Nppb*, and *Rcan1*. Among the down-regulated genes, there was *Actn2*, a previously characterized target of QKI in hESC-CM.^20^ Western blotting was performed to confirm the down-regulation of ACTN2 and the up-regulation of CAMK2D protein in the ciQKI KO hearts (Supplementary material online, *Figure S7*). To assess alternative splicing changes in the adult hearts, we detected differential exon usage with DEXSeq^24^ and found 1023 differentially spliced genes in ciQKI KO hearts. Specifically, 927 exon bins were up-regulated and 1089 exon bins were down-regulated in the ciQKI KO hearts (*Figure 4B*, Online data 2). Among the mis-spliced genes, there were multiple genes directly involved in cardiac contraction, such as *Atp2a2*, *Cacna1c*, *Cacna2d2*, *Sorbs2*, *Vcl*, *Tpm1*, or *Ttn*.

RNA-seq in the ventricles of E12.5 cQKI WT and cQKI KO mice also yielded differences in gene expression and splicing (Supplementary material online, *Figure S8A* and *B*, Online data 3 and 4). Principal component analysis on exon usage and gene expression in the embryonic and adult hearts shows that splicing changes are more distinct than gene expression changes at both timepoints (Supplementary material online, *Figure S8C* and *D*). Although there was a smaller number of differentially spliced transcripts in the E12.5 cQKI KO embryos compared with the adult ciQKI KO hearts (148 in embryos vs. 1023 in adults), a substantial proportion of these mis-spliced genes (68%) did overlap with the ones observed in the adult QKI KO hearts (*Figure 4C*). A scatterplot depicting the correlation between differentially spliced exons in embryo and adult hearts also reveals that the fold changes are larger in the adult heart (Supplementary material online, *Figure S8E*). Pathway analysis of the 1023 mis-spliced genes in the adult ciQKI KO heart showed enrichment for gene ontology ‘biological process’ terms related to muscle biology, such as ‘sarcomere organization’ and ‘striated muscle cell development’ (*Figure 4D*, Supplementary material online, *Table S6*).

To validate the splicing changes observed by RNA-seq, we performed RT–PCR in adult heart tissue (three ciQKI WT, three ciQKI HET, and five ciQKI KO) for a panel of genes involved in muscle biology. We could confirm robust splicing changes for all genes tested. As shown in *Figure 4E*, we confirm alternative splicing changes for components of myofibrils (A*ctn2*, *Ldb3*, *Nebl*, and *Ttn*), regulatory kinases (*Camk2d* and *Akap9*), calcium channels (*Cacnb1* and *Ryr2*), and transcriptional and splicing regulators (*Mbnl2*, *Mef2c,* and *Rbfox2*). Interestingly, ciQKI HET hearts did not show intermediate splicing changes, except for a subtle difference in *Ttn* and *Ryr2* splicing. Sashimi plots were generated to visualize splicing changes at the transcript level (*Figure 4F*). Without QKI, exon 4 of the Z-disc component *Ldb3* (/ZASP/Cypher) is partially skipped and exons 5–7 are aberrantly included; exons 25 and 26 of the Z-disc component nebullete (Nebl) are skipped; exons coding for Z-repeats of titin (*Ttn*) are skipped (exons 11–13);^28^ and the terminal exons of alpha-tropomyosin (*Tpm1*) are alternatively spliced.^29^ In conclusion, QKI depletion in the embryonic and adult heart leads to massive splicing changes in genes crucial for muscle biology.

### 3.4 Intronic location of the QKI-binding motifs is associated with exon skipping or inclusion

**Figure 5:**
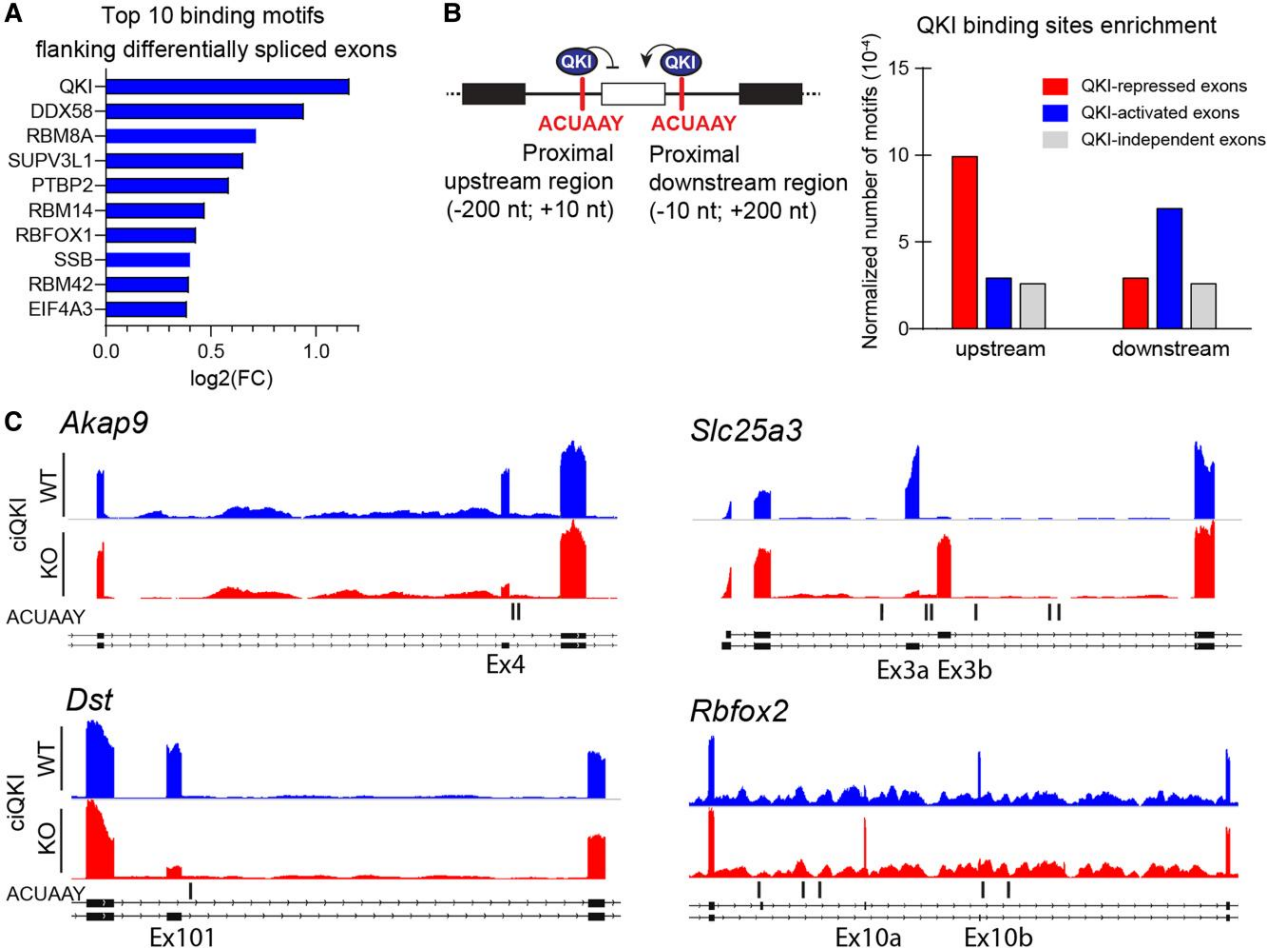
Location of the QKI-binding site determines whether QKI acts as a repressor or as an activator. (*A*) Enrichment of RNA-binding motifs surrounding differentially spliced exons in adult ciQKI KO hearts (five ciQKI WT vs. five ciQKI KO). RNA-binding motifs in an equal number of exons that were not differentially spliced were used as a reference. (*B*) Left panel: Schematic model to illustrate the positional effect of ACUAAY motifs in determining whether QKI acts as a repressor or as an activator of exon inclusion. Right panel: normalized number of ACUAAY motifs upstream and downstream of QKI-repressed, QKI-activated, and a random selection of QKI-independent exons. (*C*) Representative bedgraphs of QKI-activated and QKI-repressed exons from RNA-sequencing reads retrieved from adult ciQKI WT and KO heart (*n* = 5/group), and the location of ACUAAY motifs indicated by vertical lines under graphs.

To examine the presence and location of putative QKI-binding sites within the transcript, we performed an unbiased enrichment analysis of RBP motifs using ATtRACT, a database for RBPs and associated motifs.^30^ We compared the presence of motifs surrounding the differentially spliced exons in adult ciQKI KO hearts with motifs surrounding an equal number of exons not differentially spliced. We limited our analysis to the proximal upstream and downstream regions, encompassing 200 nt in the flanking introns and 10 nt within the exon. As anticipated, the binding motif of QKI (ACUAAY) was the most enriched sequence in the regions flanking the mis-spliced exons (*Figure 5A*, Online data 5). Interestingly, one of the other enriched sequences was the binding motif for RBFOX1. Like QKI, RBFOX1 is a splicing regulator with enriched expression in brain and striated muscle. As multiple splicing targets are shared between RBFOX1 and QKI (e.g. *Mef2c*, *Mef2d*, *Camk2d*, and *Mbnl2*),^8^ it is conceivable that they cooperate in splicing networks.

Splicing factors can have opposing effects on exon usage, depending on whether they bind to their motifs up- or downstream of the exon.^31^ Also for QKI it has been reported that the location of ACUAAY motifs within the transcript determines whether QKI acts as a repressor or as an activator of exon inclusion.^32–35^ Therefore, we quantified and compared the presence of QKI-binding sites in the proximal upstream and downstream regions of QKI-activated (exons skipped in the ciQKI KO heart), QKI-repressed (exons included in the ciQKI KO heart), and QKI-independent exons (non-differentially spliced exons) (*Figure 5B*). We observed an increase in the number of motifs *upstream* of the QKI-repressed exons and an increase in the number of motifs *downstream* of QKI-activated exons, further illustrating how the positioning of QKI relative to the exon determines whether the exon is included or excluded in the final transcript. Examples of QKI-activated exons with downstream ACUAAY motifs (exon 4 of *Akap9* and exon 101 of *Dst*) are shown in *Figure 5C*. Mutually exclusive exons 3a/3b of *Slc25a3* and 10a/10b of *Rbfox2* were found to contain the motif upstream of the QKI-repressed exon *and* downstream of the QKI-activated exon.

### 3.5 QKI is essential for the expression of muscle-specific and developmental splice isoforms

**Figure 6:**
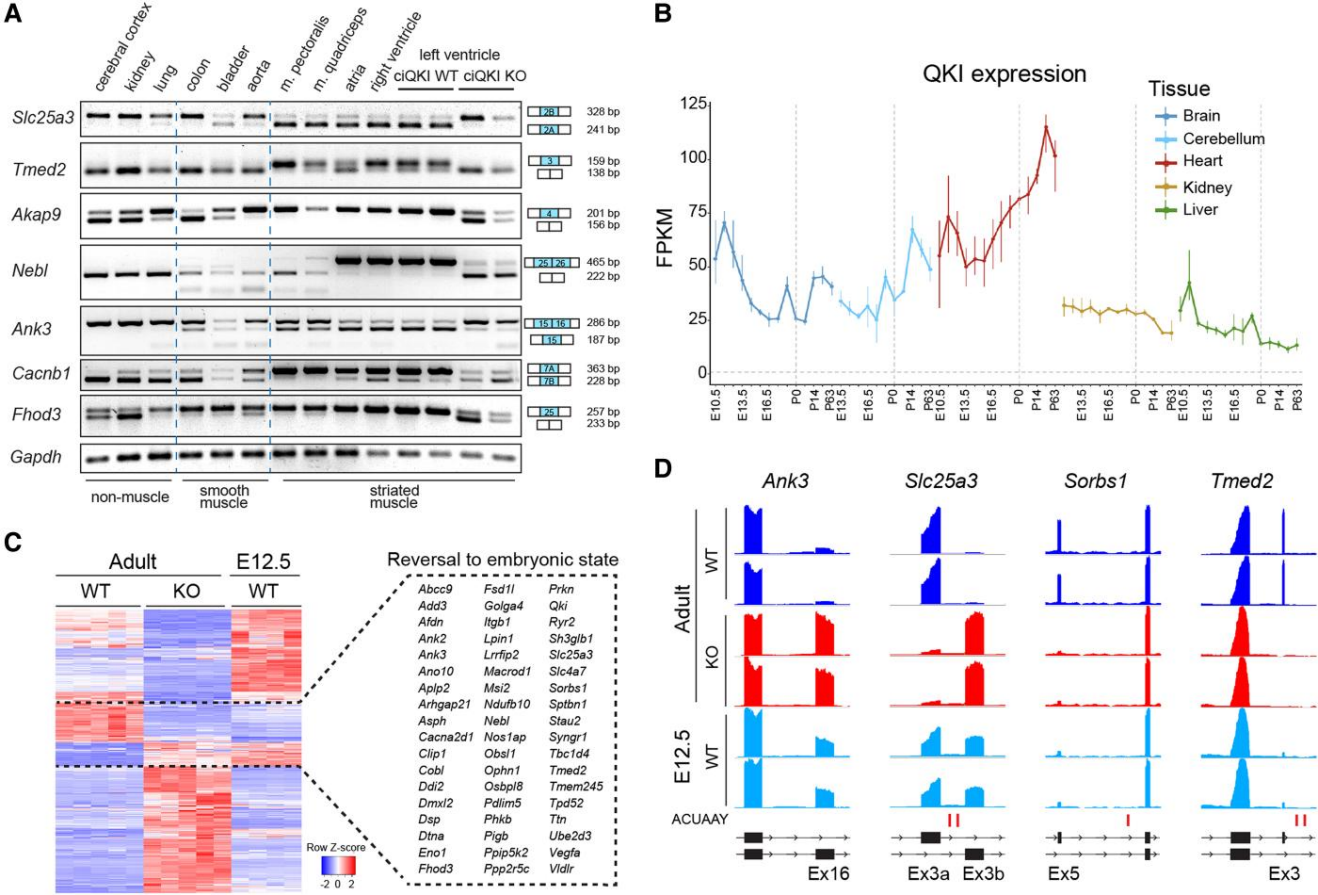
QKI depletion causes a loss of muscle-specific isoforms. (*A*) RT–PCR panel showing isoform expression across non-muscle-, smooth- and striated muscle-containing tissues in adult WT mice, and in the LV from ciQKI WT and KO mice. Tissue panels of three different mice were analysed with comparable results. (*B*) QKI expression during development across tissues in mice. Data retrieved from Cardoso-Moreira *et al*.^36^ (*C*) Heatmap showing differential exon usage in ciQKI WT and KO adult hearts (five hearts per group) and in E12.5 WT ventricles (four hearts). Gene names within the dashed box represent splice isoforms that revert to their embryonic state after loss of QKI. (*D*) Representative bedgraphs showing four examples of genes that revert to the embryonic isoform in adult ciQKI KO hearts (two mice per group shown). Predicted QKI-binding sites (ACUAAY) are depicted below bedgraphs as vertical lines.

Splice isoforms are often produced in cell-type and/or developmental-specific manner,^32^ and we wondered whether this is also the case for QKI-regulated isoforms. First, we checked whether the QKI-dependent splicing events that we observed occurred exclusively in the heart or were present in other tissues as well. To do so, we performed RT–PCR on a tissue panel of adult mice, consisting of ‘non-muscle’ tissues (cerebral cortex, kidney, and lung), tissues with a high content of smooth muscle cells (colon, bladder, and aorta), and striated muscle tissues (pectoralis muscle, quadriceps, atria, right ventricle, and left ventricle). To be able to interrogate isoform expression across multiple tissues, we selected only those QKI targets that are expressed in a ubiquitous manner. As can be appreciated from *Figure 6A*, the picture emerges that QKI depletion prevents the expression of muscle-specific isoforms. For instance, the isoform of *Slc25a3* containing exon 2A is highly enriched in striated muscle, but in the ciQKI KO hearts, this isoform is completely lost. The same holds true for the muscle isoforms of *Tmed2, Akap9*, and *Nebl*. Other QKI-dependent splicing events, like *Ank3* and *Cacnb1*, are not restricted to striated muscle types but are expressed in smooth muscle tissues as well. Interestingly, loss of QKI in the heart often results in the expression of isoforms that are normally present in the cerebral cortex (e.g. *Akap9*, *Ank3*, *Cacnb1*, *Fhod3*, *Slc25a3*, and *Tmed2*), which is remarkable since QKI is also highly expressed in the nervous system (*Figure 6A*).

Alternative splicing is a fundamental mechanism for organ development, especially in the brain and heart,^35^ tissues with relatively high levels of QKI. Interestingly, *Qki* mRNA levels increase in the heart throughout development, whereas in other tissues (brain, kidney, liver) *Qki* mRNA levels seem to decrease over time (*Figure 6B*).^36^ We used our RNA-seq data to investigate whether loss of QKI in the adult heart would lead to the expression of more embryonic splice isoforms. As such, we compared QKI-dependent splicing events that we identified in the adult hearts with the isoforms present in wild-type E12.5 hearts. As shown in the heatmap in *Figure 6C*, there were 55 genes that switched to an embryonic isoform in the absence of QKI. Four examples of QKI-dependent genes that reverted to an embryonic isoform are illustrated in the bedgraphs of *Figure 6D*. Overall, our findings illustrate that QKI controls the expression of numerous muscle- and developmental-specific splice isoforms in the heart. This puts QKI at the centre of co-ordinated splicing networks that regulates cardiac tissue identity and cardiac development.

### 3.6 QKI overexpression increases cardiomyocyte contractility

**Figure 7:**
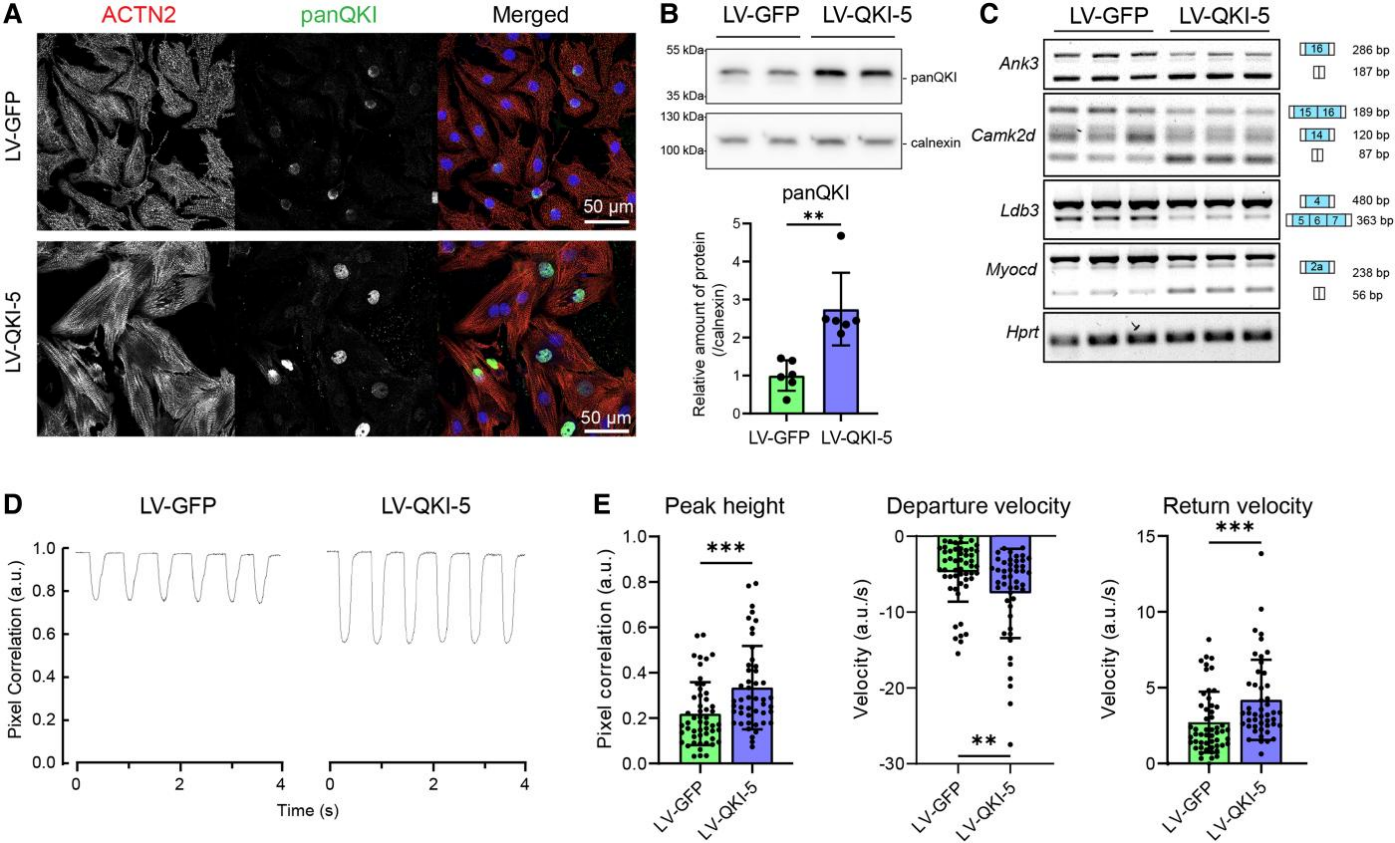
QKI overexpression in neonatal rat ventricular myocytes (NRVMs) increases the production of muscle isoforms and enhances contractility. (*A*) Representative immunocytochemistry of NRVMs 5 days after lentiviral (LV) transduction of GFP or QKI-5: ACTN2 (red), panQKI (yellow), and DAPI (blue). Scale bar: 50 µm. (*B*) QKI protein levels as measured by western blot. Unpaired *t*-test; **P < 0.01. (*C*) RT–PCR showing a shift in splicing in the QKI targets *Ank3*, *Camk2d*, *Ldb3*, and *Myocd,* 5 days after lentiviral transduction. Three wells per condition; representative results from three independent experiments are shown. (*D*) Representative contraction traces and (*E*) contraction parameters 4 days after lentiviral transduction. Departure/return velocities are the maximum velocities reached on the contraction and relaxation phase of the transient. *N* ≥ 40 cells per condition. The data represent one out of four independent experiments. Data are mean ± standard deviation. Mann–Whitney test; *P < 0.05; **P < 0.01; ***P < 0.001. All experiments were performed with a multiplicity of infection of 1.3.

Given our observation that QKI regulates a muscle-specific alternative splicing program that shapes the contractile function of cardiomyocytes, we wondered whether overexpression of QKI in NRVMs (i) would be sufficient to push the expression of adult QKI-dependent splicing isoforms and (ii) would affect contractility of cardiomyocytes. We limited our studies to QKI-5, since it is the predominant isoform in the heart and the only one with a nuclear localization signal. We made use of a bicistronic lentiviral vector to simultaneously express QKI-5 and GFP after transduction. As a control, we overexpressed GFP alone. Five days after transduction, NRVMs presented QKI protein overexpression in the nucleus (*Figure 7A*), and this was associated with an increase in cell size (Supplementary material online, *Figure S9*). QKI expression was increased approximately by 2.5-fold at the protein level (*Figure 7B*) and RT–PCRs showed a shift in splicing opposite to the one observed in the ciQKI KO hearts (*Figure 7C*). This shift towards more muscle-specific isoforms consisted of increased skipping of exon 16 in *Ank3*, higher levels of the *isoform* δ-C *of Camk2d*, more inclusion of exon 4 and skipping of exons 5–7 in *Ldb3*, and increased skipping of exon 2a in *Myocd*.

To test whether the splicing changes induced by QKI-5 overexpression affected the contractility of the NRVMs, we made use of CytoCypher, a high-throughput microscopic system able to measure cardiomyocyte contraction by calculating pixel displacement over time. We performed the measurements on Day 4 after transduction, before the increase in cell size occurs (Supplementary material online, *Figure S9*). Representative cell contraction transients of GFP- and QKI-5-transduced NRVMs are depicted in *Figure 7D*. Interestingly, QKI-5 overexpression increased the peak height of the contraction transients, and the departure and return velocities (i.e. the maximum velocities reached on the contraction and relaxation phase of the transient) (*Figure 7E*). In summary, our *in vitro* experiments indicate that QKI-5 overexpression is sufficient to increase muscle-specific splicing and enhance the contractile properties of neonatal cardiomyocytes.

## 4. Discussion

In this study, we investigated the function of QKI in the heart. We demonstrate that QKI is required in cardiomyocytes to maintain normal cardiac function during development and in the adult heart. Most strikingly, loss of QKI in adult cardiomyocytes rapidly induces heart failure, with LV dilation and severe disruption of sarcomeres, and this occurs already 7 days after deleting QKI from the heart. We show that QKI regulates the integrity and structural composition of sarcomeres, at least in part, by controlling alternative splicing of hundreds of protein-coding genes. Analysis of splicing events in ciQKI KO hearts revealed defective splicing in numerous sarcomere genes (e.g. *Nebl*, *Ttn*, *Tpm1*, *Ldb3*, and *Actn2*), cytoskeletal genes (e.g. *Ablim1*, *Ank3*, and *Dst*), calcium-handling genes (e.g. *Camk2d*, *Atp2a2*, *Ryr2*, and *Cacna1c*), and (post-)transcriptional regulators (*Mef2c*, *Myocd*, *Mbnl2*, and *Rbfox1/2*). Forced overexpression of QKI-5 in cultured neonatal cardiomyocytes directed these splicing events in the opposite direction, which led to increased contractility of cardiomyocytes.

Because QKI regulates the splicing of such a large number of developmental- and disease-relevant genes, it is currently not possible to pinpoint how each defectively spliced gene contributes to the QKI KO phenotype we observe. Nevertheless, the functional relevance of a number of these splice isoforms has been reported previously. For instance, skipping of exon 8 in *Actn2*, which we observe in QKI KO hearts, results in a premature stop codon, leading to degradation of the *Actn2* mRNA due to activation of the nonsense-mediated mRNA decay (NMD) pathway.^20^ Within *Ttn*, exons 11–13 are skipped in the absence of QKI. These exons are located in the Z-band region of TTN and alternative splicing in this region generates a variable number of Z-repeats, presumably changing the binding sites for the scaffold protein ACTN2, and thus influencing the mechanical stability of the sarcomere.^37,38^ In *Tpm1*, we observed splicing changes in the terminal exons that define TPM1 affinity for actin. Specifically, in the absence of QKI, we observed loss of striated muscle-specific exons 9a and 9b, in favour of exons 9c and 9d. Exons 9a and 9b encode for specific amino acid residues that are critical for the interaction of troponin with tropomyosin on the thin filaments.^29,39^ For CaMKIIδ, we observed a switch from the δ-C to the δ-A isoform, resulting from the inclusion of exons 15 and 16. The δ-C isoform is located in the cytoplasm, where it phosphorylates RyR2 and phospholamban, while the δ-A isoform is associated with the intercalated disc and T-tubules, where Ca^2+^ channels and activated CaMKIIδ are concentrated.^40,41^ The increased δA levels may lead to Ca^2+^-handling disturbances in QKI KO cardiomyocytes, but this warrants further investigation. The transcription factor MEF2C is a crucial regulator of myogenesis by controlling the expression of genes that encode a network of structural proteins. Here, we show that QKI is required for the expression of the MEF2C isoform containing the mutually exclusive α2 exon. The α2 isoform has potent myogenic activity, whereas the isoform containing α1 does not.^42^ It is conceivable that mis-splicing of MEF2C in the QKI KO hearts contributes to the sarcomere phenotype by reducing the transcription of muscle genes.

Two recent studies in human stem cells already uncovered a critical role for QKI in cardiomyocyte differentiation. Chen et al. employed CRISPR/Cas9 gene editing to delete QKI from human embryonic stem cells (hESC). By differentiating these stem cells into cardiomyocytes, they revealed that QKI is essential for the formation of myofibrils during cardiomyocyte differentiation.^20^ Genes involved in contractile function were found to be alternatively spliced in the absence of QKI, including *ACTN2*, *NEBL*, *ABLIM1*, *PDLIM5*, and *TTN*. Consistent with this work, Fagg *et al*.^21^ compared alternative splicing events in three cell lineages differentiated from hESCs (i.e. the endoderm, cardiac mesoderm, and ectoderm cell lineages), and showed that QKI plays a critical role in the specification of cardiac mesoderm and the formation of cardiomyocytes. One of the studied splicing targets in that study was Bridging Integrator1/Amphiphysin 2 (*BIN1*), a protein known for its role in cardiac development and myofibrillogenesis.^43,44^ In our QKI mouse models, we did not find altered splicing or gene expression of *Bin1*, neither in the adult QKI KO hearts, nor in the embryonic QKI KO hearts. This may relate to the timing of QKI deletion using the Myh6 promoter, which starts to become active from E7.5 onwards, which is after cardiac mesoderm formation. In conclusion, these human stem cell studies uncovered a role for alternative splicing in early lineage-specific gene expression and implicate QKI as a cardiac mesoderm-enriched RBP required for the differentiation of cardiomyocytes. A role for QKI in myofibril formation is, however, not limited to cardiomyocytes, as recent studies also provided evidence that QKI controls the formation of myofibrils of skeletal muscle in zebrafish^45^ and regulates a splicing program during smooth muscle development.^19,46^ In addition, QKI deficiency in muscle stem cells using a conditional QKI mouse model in combination with the Pax7-Cre driver was shown to result in loss of the myogenic progenitor cell population and muscle regeneration defects.^47^

Once assembled, cardiac sarcomeres require constant maintenance as the continuous contractions of the heart are accompanied by mechanical stress, which in turn predisposes proteins to misfolding and aggregation.^48^ Maintenance of sarcomeres is a complex process, in which new proteins are constantly incorporated into sarcomeres to replace old ones without compromising mechanical function. The half-lives of sarcomere proteins in the heart are estimated in the order of days to weeks.^48^ Our finding that sarcomeres in the adult heart degenerate already 1 week after QKI depletion suggests that sarcomere maintenance is compromised by the incorporation of aberrant protein isoforms. This is particularly relevant, as there is evidence that QKI expression is lost during multiple forms of heart disease. This was shown in a mouse model of doxorubicin-mediated cardiotoxicity,^18^ in a rat model of ischaemia/reperfusion,^16^ in the diabetic mouse heart,^49^ and in human heart failure.^18^ Accordingly, reduced levels of QKI may very well contribute to the disease mechanism of sarcomere dysfunction and decreased contractility in heart failure. In this light, it has been shown that more than 1000 transcripts express aberrant isoforms in ischaemic and dilated cardiomyopathies, indicating that alternative splicing is a highly regulated process and an important layer of gene regulation during pathological remodelling of the heart.^50,51^ It will be very interesting to see whether therapeutic up-regulation of QKI using gene therapy in acquired heart disease will be able to shift splicing towards more cardioprotective isoforms and improve cardiac remodelling and function. This is strengthened by our observation that QKI overexpression is sufficient to increase contraction and relaxation velocities of NRVMs. Moreover, Gupta et al. recently provided evidence that therapeutic overexpression of QKI is beneficial in a mouse model of doxorubicin-mediated heart failure.^18^ They showed that adeno-associated virus serotype 9 (AAV9)-mediated overexpression of QKI attenuates apoptosis and atrophy of cardiomyocytes in this model. Alternative splicing of sarcomere genes was not investigated in this study, instead certain circRNA molecules were identified as mediators in the observed anti-apoptotic effect. A role for QKI in cardiomyocyte apoptosis was also reported in a study by Guo et al., who showed that QKI inhibits ischaemia/reperfusion-induced cardiomyocyte apoptosis by regulating *Foxo1* mRNA stability.^16^ Notably, we did not find altered expression or splicing of *Foxo1* mRNA in our QKI KO datasets.

It has become evident that alternative splicing occurs in co-ordinated networks, rather than in isolated events. Several splicing factors, including RBM20,^3^ RBM24,^9^ SRSF10,^52^ ASF/SF2,^41^ and RBFOX1,^8^ were shown to regulate alternative splicing of a large number of transcripts in the developing and/or adult heart. By comparing their splicing targets, the picture emerges that these splicing factors regulate a distinct, but overlapping set of transcripts. For instance, the switch in CaMKIIδ, from the δ-C to the δ-A isoform that we identified in QKI KO hearts, has also been found in RBM20 and ASF/SF2 KO mice.^3,41^ The inclusion of the mutually exclusive exons 3a/3b of the mitochondrial phosphate carrier *Slc25a3* is regulated by QKI and RBM24.^9^ Splicing of TTN is regulated by RBM20 and by QKI; however, these factors control splicing of different exons. Whereas RBM20 is responsible for the inclusion of I-band exons, a region of TTN that is crucial for the elastic property of sarcomeres, QKI regulates the inclusion of Z-band exons, a region that is thought to be important for the mechanical stability of the sarcomere.^37^ We found that one of the most enriched sequences in the flanking introns of QKI-regulated exons is the binding motif for RBFOX1, a splicing regulator that is also enriched in brain and striated muscle. As multiple splicing targets are shared between RBFOX1 and QKI (e.g. *Mef2c*, *CamkIId*, and *Mbln2*), it is conceivable that QKI and RBFOX1 engage in a complex regulatory network of alternative splicing.^8^ The full extent of how QKI cooperates and competes with other splice factors is yet to be determined. Finally, our observation that QKI also regulates alternative splicing of *Rbfox1* itself adds another layer of complexity and warrants further exploration.

In conclusion, we are the first to show that QKI acts as a major regulator of striated muscle identity in the cardiomyocytes of the adult heart, by directing the expression of muscle-specific isoforms of numerous pre-mRNAs. This extends previous studies showing a role for QKI in cardiomyocyte differentiation during the transition from cardiac progenitors to early cardiomyocytes^20^ and in the specification of cardiac mesoderm.^21^ Future studies are needed to investigate whether QKI dysregulation contributes to the mechanism of contractile dysfunction in heart disease. Specifically, the fact that QKI is down-regulated in human DCM,^18^ and our observation that forced overexpression of QKI enhances cardiomyocyte contractility, may open avenues for therapeutic strategies to adapt cardiac isoform expression and improve cardiac function in heart failure patients.

## Authors’ contributions

P.M.A. and E.E.C. contributed to the experimental design, writing, and editing of the manuscript. P.M.A., S.A., E.N.S., I.v.d.M., and E.E.C. contributed to the acquisition of data, analysis, and interpretation. L.C.O., A.C.E., S.R., D.W.D.K., and V.M.C. provided experimental resources. S.R., D.W.D.K., V.M.C., Y.M.P., and E.E.C. revised the intellectual content of the manuscript. Y.M.P. and E.E.C. supervised the project. All authors read and approved the manuscript.

## Supplementary Material

cvad007_Supplementary_Data

## Data Availability

Raw RNA-sequencing data are available at NCBI BioProject, under ID number PRJNA831665.

## Notes

Translational perspective Alternative splicing generates protein isoforms to maintain mechanical, structural, and metabolic properties of cardiomyocytes. We are the first to show that QKI is one of the essential splicing factors in the adult heart. During heart failure, alternative splicing of numerous genes is altered, thereby affecting cardiac function. Recent observations that QKI expression is down-regulated in hearts of heart failure patients indicate that loss of QKI-mediated processes contributes to decreased sarcomere organization in these patients. Modulation of QKI activity may serve as a future therapeutic strategy to adapt cardiac isoform expression and improve cardiac function in heart failure patients.
