# Supplementary material for: The RNA-binding protein QKI governs a muscle-specific alternative splicing program that shapes the contractile function of cardiomyocytes
Source: Cardiovasc Res. 2023 Jan 11;119(5):1161–74. doi: 10.1093/cvr/cvad007 (PMC10202634; doi:10.1093/cvr/cvad007)

**The RNA-binding protein QKI governs a muscle-specific alternative splicing program that shapes the contractile function of cardiomyocytes**

Pablo Montañés-Agudo^1^, Simona Aufiero^1,2^, Eva N. Schepers^1^, Ingeborg van der Made^1^, Lucia Cócera-Ortega^1^, Auriane C. Ernault^1^, Stéphane Richard^3^, Diederik W.D. Kuster^4^, Vincent M. Christoffels^5^, Yigal M. Pinto^1^, Esther E. Creemers^1^

Amsterdam UMC, location University of Amsterdam, Departments of ^1^Experimental Cardiology, ^2^Clinical Epidemiology, Biostatistics and Bioinformatics, and ^5^Medical Biology, Meibergdreef 9, Amsterdam, The Netherlands

^3^Segal Cancer Center, Lady Davis Institute for Medical Research and Gerald Bronfman Department of Oncology and Departments of Biochemistry, Human Genetics and Medicine, McGill University, Montréal, QC H3T 1E2, Canada.

^4^Amsterdam UMC, location Vrije Universiteit Amsterdam, Physiology, De Boelelaan 1117, Amsterdam, Netherlands

**Short running title:** QKI function in the heart

**Corresponding author:**

Esther E. Creemers, PhD

Experimental Cardiology, Room K2-104-2

Amsterdam UMC, location University of Amsterdam

Meibergdreef 15

1105AZ Amsterdam

The Netherlands

+31-20-5663262

[e.e.creemers@amsterdamumc.nl](mailto:e.e.creemers@amsterdamumc.nl)

**Supplemental Methods**

**Echocardiography and ECG recordings**

Echocardiography and ECG recordings were performed 1 week after the last tamoxifen injection on mice sedated with 4% isoflurane and maintained in anesthesia by a mixture of O2 and 2.5% isoflurane. LV function and dimensions were measured by transthoracic two-dimensional echocardiography using a Vevo 770 Ultrasound (Visual Sonics) equipped with a 30-MHz linear array transducer. M-mode tracings in parasternal short axis view at the height of the papillary muscle were used to measure LV internal diameter at end-systole and end-diastole. For ECG acquisition, electrodes were placed at the right (R) and left (L) armpit and the left groin (F). A reference electrode was placed at the right groin. ECGs were recorded (Biosemi, Amsterdam, the Netherlands; sampling rate 2048 Hz, filtering DC 400 kHz (3 dB)) for a period of 5 min. After the recordings, sedated mice were euthanized by cervical dislocation.

**cDNA synthesis and RT-PCRs**

For cDNA synthesis, 500 ng of total RNA were treated with DNAse I (Invitrogen, Ref 18068-015, Waltham, MA, USA) and retrotranscribed into cDNA with random hexamers (Invitrogen, Ref N8080127) and Superscript II (Invitrogen, Ref 18064-014). qRT-PCRs were performed on a Lightcycler 480 (Roche, Mannheim, Germany) using SYBR green I Master Mix (Roche, Ref 04887352001). Data was analyzed using LinRegPCR software. RT-PCRs were performed in a 25 µL reaction containing 5 ng cDNA, 1M Betaine, 1x Buffer B2, 2.5 mM MgCl2, 200 µM dNTPs, 0.4 µM forward primer, 0.4 µM reverse primer and 0.05 U/µ HOT FIREpol® DNA polymerase (Solis Biodyne). Primer sequences are found in **Table S1.** The thermal cycling protocol included an initial denaturation at 95 °C for 15 min to activate the HOT FIREPol® DNA polymerase, followed by 35 amplification cycles of denaturation at 95 °C for 30 s, annealing at 60 °C for 30 s, extension at 72 °C for 45 s, and 5 minutes of final extension at 72°C. *Slc25a3* PCR product was digested with the restriction enzyme XbaI (Roche) to distinguish exon 2A from 2B.

**Western Blotting**

For protein isolation, flash-frozen tissue was homogenized by MagNA Lyser Green Beads (Roche) in RIPA buffer (50 mM Tris-HCl pH 8, 150 mM NaCl, 1% NP-40, 0.2% sodium deoxycholate, 0.1% SDS, 1 mM Na_3_VO_4_, 1 mM PMSF) supplemented with protease inhibitor cocktail (Roche). Protein concentration was measured by BCA assay (Pierce). Western blotting was performed following standard protocols. Proteins were separated by SDS-PAGE, transferred to PVDF membranes (Bio-Rad), block in TBST 4% milk and incubated with primary antibodies overnight at 4°C. Membranes were subsequently incubated with HRP-conjugated secondary antibodies for 1 hour at room temperature. Western blots were developed with ECL prime western blotting detection agent (Amersham Biosciences) in ImageQuant LAS 4000 (GE 194 Healthcare Life Sciences). Western blots were quantified using Fiji (ImageJ). Antibody references are listed in **Table S2.**

**Histology**

Hearts and embryos were fixed in 4% paraformaldehyde and embedded in paraplast. Paraplast blocks were cut in 5 µm sections and slides were stained with hematoxylin&eosin using standard techniques. For immunohistochemistry, sections were deparaffinized, rehydrated in a series of ethanol, and boiled for 15 min in antigen unmasking solution (Vector laboratories Ref H3300, Burlingame, California, USA) in a pressure-cooker. After cell membrane permeabilization with 0.5% Triton-X-PBS and blocking with 4% BSA-PBS, samples were incubated with primary antibodies overnight at 4°C and stained with secondary antibodies and DAPI for 2 hours at room temperature. Pictures were taken on a confocal microscope (Leica SP8, Leica Microsystems, Germany). Antibodies and their dilutions are listed in **Table S2.**

Transmission electron microscopy was performed by the Cellular Imaging Facility of the Amsterdam UMC. In short, LV tissue was prefixed in 1% glutaraldehyde, 4% paraformaldehyde in 0.1M sodium cacodylate and processed for electron microscopy. Images were acquired with a FEI technai 12 transmission electron microscope. Sarcomere organization was scored manually by taking random pictures of sarcomere areas and scoring for 2 different categories - organized or aberrant (**Fig S6B**) - in 3 hearts per group (4 areas per heart) on a blinded basis.

## RNA sequencing

Library preps were made with the Kapa RNA Hyperprep with RiboErase (Roche) and sequenced on a NovaSeq platform (NovaSeq S4.300; flow cell type PE150, 2 x 150nt). Quality control of FASTQ files was performed using FastQC (Babraham Bioinformatics). Trimmomatic (version 0.351) was used to remove adapters and low quality bases, using a Phred score cutoff of 30 while discarding reads with a length below 75 bases. All samples had a sequencing depth between 178-385 million reads per sample and all samples passed quality control. Paired-end reads were then aligned against the mouse genome. Gene ontology enrichment analysis was done using the online tool PANTHER^2^. Genes expressed in mouse heart with TPM > 0.5 were used as background.

**RBP analysis binding sites analysis**

RBP analysis was performed on the differentially spliced exons identified. Exon bins with absolute log2 fold change cut-off ≥1, adjusted P-value cutoff ≤ 0.05 and which corresponding genes are expressed with TPM value ≥ 1. Furthermore, only exon bins whose genomic coordinates matched with the genomic coordinates of an exon reported in the genome annotation files were retained. After filtering a total of 1126 differentially expressed exons (604 upregulated exons and 522 downregulated exons) were used as foreground set. An equal number of not differentially spliced exons were used as background set. Then the table containing the collected exons was adapted to work with module 11 of the R Bioconductor package circRNAprofiler^3^ to retrieve the target sequences. The following settings were used: lIntron = 200, lExon = 9, type = ie. Then the target sequences were scanned for the presence of RBP binding motifs by using module 12 with the following settings: width = 6, database = ATtRACT, rbp = TRUE, reverse = FALSE. QKI motif was included in motifs.txt (as specified by circRNAprofiler). Raw and normalized counts are reported in **Online Data 5**. Normalization was performed by dividing the number of occurrences of each motif by the total length of the target sequences. The same analysis was performed this time only for the differentially spliced exons. In detail the upregulated exons were as used as foreground set while the downregulated exons were used as background set. The QKI binding sites were then retrieved for the upstream and the downstream regions of the corresponding exons.

**Lentiviral constructs**

Expression plasmid pLV-QKI-5 was a gift of Dr. van der Veer (Leiden, The Netherlands). QKI-5 sequences were subcloned in a bicistronic pLV-CMV-x-IRES-GFP lentiviral vector. For lentivirus production, HEK293T cells were transfected with pLV-CMV-empty-IRES-GFP or pLV-CMV-QKI-5-IRES-GFP, in combination with the packing vectors pVSVG, pMDL and pRSV. Lentiviral particles were collected from the supernatant. Transducing units were calculated by transduction of HEK293T cells and quantification of GFP positive cells by flow cytometry.

**Neonatal rat ventricular cardiomyocytes (NRVMs) isolation**

NRVMs from 0–2 day old Wistar rats (Janvier Labs, Le Genest-Saint-Isle, France) were isolated as described previously^4^. In short, pups were anesthetized by isoflurane and hearts were excised after decapitation. Ventricles were cut into small pieces, which were incubated overnight in a rotating platform at 4 °C in HBSS (Gibco, Ref 14170-088, Paisley, UK) containing 1 mg/ml trypsin (USB, Ref 22720, Cleveland, OH, USA). The next day, cells were dissociated using 1 mg/ml collagenase type 2 (Worthington, Ref LS004177, Lakewood, NJ, USA) in HBSS. Cells were collected and resuspended in TUNG medium - M199 culture medium (Gibco, Ref 31150-022), 1% HEPES (Gibco, Ref 15630-080), 1% NEAA (Gibco, Ref 11140-050), 2 mg/L vitamin B12 (Sigma, Ref V2876, St. Louis, MO, USA), 3.5 g/L glucose, 1% penicillin/streptomycin (Gibco, Ref 15140-122) - supplemented with 10% FBS (Biowest, Ref S1810-500, Riverside, MO, USA). The cell suspension was pre-plated to separate myocytes from fibroblasts. After 2 hours, non-adhered myocytes were collected and counted with a LUNA-II Automated Cell Counter (Logos Biosystems, Anyang, South Korea).

**NRVM transduction**

NRVMs were plated on 6-well plates coated with fibronectin (Corning, Ref 356008, Bedford, MA, USA) in TUNG medium supplemented with 10% FBS containing lentiviral particles (MOI 1.3). 24h after lentiviral transduction, NRVMs were washed twice with HBBS and medium was replaced with TUNG 2% FBS supplemented with 10 nM of the cytostatic Ara-C (Sigma, ref C-6645, St Louis, MO, USA) to inhibit fibroblast proliferation.

**NRVM immunohistochemistry and staining**

NRVMs were seeded on fibronectin-coated glass coverslips on 24-well. Cells were fixed in 4% PFA, permeabilized with PBS 0,1% Triton X-100, blocked in 4% goat serum and incubated overnight with the primary antibodies in a wet chamber at 4°C. Secondary antibodies and DAPI (Molecular probes) were incubated for 1h at room temperature. For cell membrane staining, non-permeabilized fixed cells were incubated with WGA-488 and DAPI for 15 min at room temperature. Images were acquired with a Leica TCS SP8 X unit mounted on a Leica DMI6000 inverted microscope. Antibody references are found in **Table S2**.

## Quantitative analysis of NRVM contraction

NRVMs were seeded on μ-Plate 24 Well Black ID 14 mm ibiTreat (Ibidi GmbH, Germany) coated with fibronectin (Corning, Ref 356008, Bedford, MA, USA). Cell contraction transients were recorded using the Pixel Correlation method with a CytoCypher Multicell High Throughput System (CytoCypher BV, Netherlands). During acquisition, cells were maintained in a climate control chamber (37ºC, 5% CO_2_, 80% humidity) and paced at 1.5 Hz. Contraction transients were analyzed with CytoCypher Cytosolver (CytoCypher BV, Netherlands).

**Supplemental figures**

**Figure S1.** Embryonic measurements. (A) The length of the dorsal-ventral axis was calculated by measuring the distance between the spine and the apex of the heart using ImageJ. Scale bar: 1 mm. (B) Area of neural tube (blue) and ventricular myocardium (red) were calculated with ImageJ as shown. The sections shown in (A) and (B) are derived from a representative cQKI WT E12.5 embryo. The measurement of each embryo is the average of 3 sections. N = 3 embryos per group. Unpaired t-test; ** p<0.01.

**Figure S2.** Representative immunocytochemistry cQKI WT and KO embryos at E10.5, E12.5 and E14.5, stained for cardiac troponin I (CTNI) and panQKI. Scale bar: 500 µm. Representative images of 3 embryos per group and per timepoint are shown.

**Figure S3.** Representative ECG recordings of ciQKI WT, HET and KO mice, 7 days after the last tamoxifen injection does not reveal any abnormality. See **Table S5** for quantification of heart rate, PR interval, QRS interval and QTc times.

**Figure S4. Knock-out of QKI induces a rapid loss of ACTN2 in cardiomyocytes.** Representative immunocytochemistry for ACTN2 and panQKI in ciQKI WT, HET and KO LV tissue. Scale bar: 40 µm and 10 µm. Representative images of 3 hearts per group are shown.

**Figure S5. QKI knock-out induces loss of TTN organization in the sarcomeres.** Representative immunocyctochemistry for TTN and panQKI in ciQKI WT and ciQKI KO hearts. Scale bar: 30 µm.

**Figure S6.** **Transmission Electron Microscopic images of ciQKI WT and ciQKI KO myofibrils in the adult hearts**. (A) Electron microscopy images of ciQKI WT and KO myofibrils illustrating glycogen deposits (white arrows) near disrupted myofibrils in ciQKI KO. Scale bar: 500 nm. (B) Example of sarcomeres scored as normal or aberrant, derived from a ciQKI WT (normal sarcomere) and a ciQKI KO (aberrant sarcomeres) respectively. Scale bar: 1 µm. Representative images of 3 hearts per group are shown.

**Figure S7. ciQKI KO hearts express lower levels of ACTN2 and higher levels of CAMK2D at the protein level.** Western Blot showing the changes in QKI, ACTN2 and CAMK2D in left ventricles of 3 ciQKI WT, 3 ciQKI HET and 5 ciQKI KO mice, 7 days after the last tamoxifen injection. In the lower panel, protein quantifications of the blot are shown, as well mRNA levels of *Actn2* and *Camk2d* (derived from the RNA-seq). Data are mean ± standard deviation. One-way ANOVA followed by Tukey’s multiple comparison test; **** adjusted p< 0.0001; ** adjusted p<0.01; * adjusted p<0.05.

**Figure S8. Gene expression and differential exon usage in embryonic hearts at E12.5** (A) Differential gene expression and (B) differential exon usage of 4 cQKI WT (*Qki^wt/wt^, Myh6-Cre^tg^*) and 4 cQKI KO (*Qki^fl/fl^, Myh6-Cre^tg^*) E12.5 hearts. (C) Principal component analysis of gene expression and (D) exon usage in embryos (E12.5, n=4 per group) and adult hearts (n=5 per group). QKI WT or KO genotypes are indicated by different colors. (E) Scatter plot showing the correlation in differential exon usage in embryos and adults. Only exon bins with adj-p ≤ 0.05 in the cQKI KO embryos were included.

**Figure S9. Cell surface measurements of NRVMs after QKI-5 overexpression.** (A) Representative WGA-488 stainings, 4 days after lentiviral transduction of GFP or QKI5. Scale bar: 50 µm.(B) Cell surface measurements at different time points. Each dot represents a cell, each time point corresponds to a different isolation. n ≥ 33 cells per condition per time-point. Man-Whitney test; *** p < 0.001, * p < 0.05.

**Supplemental tables**

**Table S1. Primer sequences**

| **Primer** | **Sequence (5’ – 3’)** | **Analysis** | **Species** |
| --- | --- | --- | --- |
| Qki 2lox Fw | ACAGAGGCTTTTCCTGACCA | Genotyping | Mouse |
| Qki 1lox Fw | CCTGGAATGGTGCTTTCCTA | Genotyping | Mouse |
| Qki 2/1lox Rv | TTCAGAACCCCCACATTACC | Genotyping | Mouse |
| Myh6-Cre Fw | ATGACAGACAGATCCCTCCTATCTCC | Genotyping | Mouse |
| Myh6-Cre Rv | CTCATCACTCGTTGCATCATCGAC | Genotyping | Mouse |
| Gapdh ex 7 Fw | GGTGGACCTCATGGCCTACA | qPCR | Mouse |
| Gapdh ex 7 Rv | CTCTCTTGCTCAGTGTCCTTGCT | qPCR | Mouse |
| Hprt ex 6 Fw | GCTTGCTGGTGAAAAGGACCTCTCGAAG | qPCR | Mouse |
| Hprt ex 8Rv | CCCTGAAGTACTCATTATAGTCAAGGGCAT | qPCR | Mouse |
| Tbp ex 4 Fw | TATGACCCCTATCACTCCTG | qPCR | Mouse |
| Tbp ex 7 Rv | TTCTTCACTCTTGGCTCCTGT | qPCR | Mouse |
| Qki ex 2 Fw | GGAGTGCAGAATTGCCTG | qPCR | Mouse |
| Qki ex 3 Rv | CTAGGTCCAAGGATTCTCC | qPCR | Mouse |
| Eef1e1 Fw | TCCAGTAAAGAAGACACCCAGA | qPCR | Rat |
| EeF1e1 Rv | GACAAAACCAGCGAGA | qPCR | Rat |
| Gapdh Fw | GGTGGACCTCATGGCCTAC | qPCR | Rat |
| Gapdh Rv | CTCTCTTGCTCTCAGTATCCTTGCT | qPCR | Rat |
| Hprt Fw | TGACTATAATGAGCACTTCAGGGATTT | qPCR | Rat |
| Hprt Rv | CGCTGTCTTTTAGGCTTTGTACTTG | qPCR | Rat |
| Ablim1 ex 17 Fw | GTTCCAGATCAAGGGATCAACATTTACCG | Splicing | Mouse |
| Ablim1 ex 19 Rv | CTTCATCAACTGTTCTTCTTGAAGCTGCC | Splicing | Mouse |
| Actn2 ex 7 Fw | GCCTTGGACTCTGTGCCCTCATC | Splicing | Mouse |
| Actn2 ex 9 Rv | CTCAGGCGTCCTGTTCTCCAGC | Splicing | Mouse |
| Akap9 ex 2 Fw | GCTCGGAAAGTTCTCAGAGGGTAGAC | Splicing | Mouse |
| Akap9 ex 4 Rv | GAAGACAGGTCTGACTGGACTGAGC | Splicing | Mouse |
| Ank3 ex 15 Fw | GGCATGAGGATGTAGCTGCGTTC | Splicing | Mouse, rat |
| Ank3 ex 18 Rv | GCGATGTGCAGTGGTGTATAGCC | Splicing | Mouse, rat |
| Cacnb1 ex 5 Fw | GGTCAAACTGGACAGCCTTCGTCTG | Splicing | Mouse |
| Cacnb1 ex 8 Rv | GACGGTCCCACCAGGATGATGG | Splicing | Mouse |
| Camk2d ex 14 Fw | CAGCCAAGAGTTTATTGAAGAAACCAGATGGG | Splicing | Mouse |
| Camk2d ex 17 Rv | CTTTCACGTCTTCATCCTCAATGGTGGTG | Splicing | Mouse |
| Fhod3 ex 24 Fw | CCTTATGCAATTCGAGAGGTGAACATCAAC | Splicing | Mouse |
| Fhod3 ex 25 Rv | GCTGCATCCTCAGCATAGCTCAG | Splicing | Mouse |
| Ldb3 ex 4 Fw | GCCTATTCCCATCTCCACGA | Splicing | Mouse, rat |
| Ldb3 ex 7 Rv | GCCTGGTACACAGGAGAGGC | Splicing | Mouse, rat |
| Nebl ex 24 Fw | GCTTCACTCCCGTTGTGGATGATC | Splicing | Mouse |
| Nebl ex 27 Rv | GCATTGACCTCATGGACGACACG | Splicing | Mouse |
| Mbnl2 Fw 7 | CCAGCAGGCTCTGACCAGTG | Splicing | Mouse |
| Mbnl2 Rv 9 | AGACAGTGGCGGACGTAGCG | Splicing | Mouse |
| Mef2c ex 6 Rv | TCTCAAAGCTGGGAGGTGGAACAG | Splicing | Mouse |
| Mef2c ex α1 Fw | GCCCTGAGTCTGAGGACAAGTACAG | Splicing | Mouse |
| Mef2c ex α2 Fw | CCGATCCTGACTCCTCTTATGCACTC | Splicing | Mouse |
| Rbfox2 ex 9 Fw | CGGACAGTGTATGGTGCAGTCC | Splicing | Mouse |
| Rbfox2 ex 13 Rv | GCTGTAGCCACCTCGGTACAAAC | Splicing | Mouse |
| Ryr2 ex 74 Fw | GACTTGCCAAACAGGACAGAAGACC | Splicing | Mouse |
| Ryr2 ex 76 Rv | CTCTGCTTAGAGAGTAGTTTGTGCCAC | Splicing | Mouse |
| Slc25a3 ex 1 Fw | GTTCTCGTCCGTAGCGCACC | Splicing | Mouse |
| Slc25a3 ex 3 Fw | GCCATCTTCTTTCAGTGTAATGGAGAATCC | Splicing | Mouse |
| Tmed2 ex 2 Fw | CACCATTGACATTGGGGAGGCTC | Splicing | Mouse |
| Tmed2 ex 3 Rv | CGGACTTCCATGTACTCCTGTTCGTG | Splicing | Mouse |
| Ttn ex 10 Fw | GCATTTGTACCAAAGGTAGTGATCTCCG | Splicing | Mouse |
| Ttn ex 14 Rv | CACAGCAGCTACAACTGTTGCCAC | Splicing | Mouse |
| Myocd ex 2 Fw | AGTTACGGCTTCAACAGAGAAGGACCCAGG | Splicing | Mouse, rat |
| Myocd ex 5 Rv | TTGAGCTTCATCTGAGCAGTTGGAATGG | Splicing | Mouse, rat |
|  |  |  |  |
|  |  |  |  |

**Table S2. Antibodies**

| **Antibody** | **Dilution** | **Reference** |
| --- | --- | --- |
| Anti-α-actinin (ACTN2) | 1:500 (IF) | Epitomics 2310-1 |
| Anti-α-actinin (ACTN2) | 1:10000 (WB) | Sigma A7811 |
| Anti-calnexin | 1:10000 (WB) | Milipore 208880 |
| Anti-CaMKII | 1:200 (WB) | Santa Cruz sc-9035, M-176 |
| Anti-desmin | 1:200 (IF) | ThermoFisher PA5-16705 |
| Anti-panQKI | WB (500), 1:50 (IF) | Sigma-Aldrich MAB N147/6 |
| Anti-titin (PEVK- IG junction) | 1:100 (IF) | Myomedix TTN-5 |
| Anti-rabbit-HRP | 1:10000 (WB) | Amersham NA9340V |
| Anti-mouse-HRP | 1:10000 (WB) | Amersham NA9310V |
| Donkey anti-rabbit-488 | 1:250 (IF) | Invitrogen A-21206 |
| Donkey anti-mouse-647 | 1:250 (IF) | Invitrogen A-31571 |
| Goat anti-rabbit-564 | 1:250 (IF) | Invitrogen A-11008 |
| Goat anti-mouse-647 | 1:250 (IF) | Invitrogen A-21235 |

**Table S3. Postnatal genotype distribution from *Qki* floxed *Myh6-Cre* mice (*Qki^fl/wt^;Myh6-Cre^tg^* x *QkiI^fl/wt^; Myh6-Cre^-^)***

| Genotypes | Qki^wt/wt^  Myh6-Cre^-^ | Qki^fl/wt^  Myh6-Cre^-^ | Qki^fl/fl^  Myh6-Cre^-^ | Qki^wt/wt^  Myh6-Cre^tg^ | Qki^fl/wt^  Myh6-Cre^tg^ | Qki^fl/fl^  Myh6-Cre^tg^ |
| --- | --- | --- | --- | --- | --- | --- |
| % observed (n) | 20%  (23) | 34%  (40) | 13%  (15) | 9%  (10) | 25%  (30) | 0%  (0) |
| % expected | 12.5% | 25% | 12.5% | 12.5% | 25% | 12.5% |

Chi-squared test (χ2) was used to calculate the statistical difference between observed and expected genotypes in the offspring. p-val χ2 = 1,63E-04.

**Table S4. Genotype distribution in E10.5-E14.5 embryos of the *Qki* floxed *Myh6-Cre* line (*Qki^fl/wt^;Myh6-Cre^tg^ x Qki^fl/wt^; Myh6-Cre^-^*)**

| Genotypes | Qki^wt/wt^  Myh6-Cre^-^ | Qki^fl/wt^  Myh6-Cre^-^ | Qki^fl/fl^  Myh6-Cre^-^ | Qki^wt/wt^  Myh6-Cre^tg^ | Qki^fl/wt^  Myh6-Cre^tg^ | Qki^fl/fl^  Myh6-Cre^tg^ |
| --- | --- | --- | --- | --- | --- | --- |
| % observed (n) | 15.5 % (15) | 25.8  (25) | 9.3%  (9) | 11.3 % (11) | 26.8%  (26) | 11.3%  (11) |
| % expected (n) | 12.5%  (12) | 25.0%  (24) | 12.5%  (12) | 12.5%  (12) | 25.0%  (24) | 12.5%  (12) |

Chi-squared test (χ2) was used to calculate the statistical difference between observed and expected genotypes in the offspring. p-val = 0,81.

**Table S5. Cardiac parameters of ciQKI WT, HET and KO mice 7 days after the last tamoxifen injection.**

|  | **ciQKI WT**  (n=5) | **ciQKI HET**  (n=6) | **ciQKI KO**  (n=9) | **ANOVA**  **p-value** |
| --- | --- | --- | --- | --- |
| BW (g) | 23.24 ± 2.37 | 24.62 ± 4.48 | 21.36 ± 3.48 | 0.25 |
| HW (mg) | 109.6 ± 13.89 | 126.8 ± 17.37 | 105.8 ± 37.42 | 0.37 |
| HW/TL (mg/mm) | 6.56 ± 0.78 | 7.52 ± 0.91 | 7.01 ± 1.04 | 0.27 |
| HW/BW (mg/g) | 4.72 ± 0.41 | 5.23 ± 0.79 | 5.42 ± 0.61 | 0.18 |
| LW/TL(mg/mm) | 7.21 ± 1.11 | 8.08 ± 2.04 | 7.74 ± 1.65 | 0.70 |
| LVID; d (mm) | 4.75 ± 0.25 | 4.64 ± 0.41 | 4.85 ± 0.41 | 0.61 |
| LVID; s (mm) | 3.79 ± 0.32 | 3.68 ± 0.47 | 4.33 ± 0.34***^#^** | 0.01 |
| LVPW;d (mm) | 0.59 ± 0.14 | 0.63 ± 0.08 | 0.56 ± 0.14 | 0.61 |
| LVPW;s (mm) | 0.69 ± 0.12 | 0.74 ± 0.09 | 0.68 ± 0.14 | 0.66 |
| FS (%) | 19.94 ± 6.24 | 20.69 ± 6.96 | 10.27 ± 4.81***^#^** | 0.005 |
| EF(%) | 40.39 ± 11.20 | 41.67 ± 12.27 | 22.12 ± 9.83***^#^** | 0.005 |
| Heart Rate (BPM) | 501.26 ± 39.94 | 497.50 ± 50.03 | 515.86 ± 28.61 | 0.69 |
| PR Interval (ms) | 39.93 ± 4.60 | 46.41 ± 7.24 | 42.42 ± 5.65 | 0.24 |
| QRS Interval (ms) | 9.55 ± 0.64 | 10.02 ± 1.58 | 9.02 ± 1.01 | 0.31 |
| QTc (ms) | 52.26 ± 4.53 | 53.67 ± 4.38 | 47.09 ± 10.61 | 0.36 |

Data are presented as mean ± standard deviation. Statistical significance was calculated with one-way ANOVA test followed by multiple comparison Fisher’s LSD test. ***** p-val < 0.05 against ciQKI WT; **^#^** p-val < 0.05 against ciQKI HET. BW, body weight; HW, heart weight; TL, tibia length; LW, lung weight; LVID, left ventricular internal diameter; d, diastolic; s, systolic; LVPW, left ventricular posterior wall thickness; FS, fractional shortening; EF, ejection fraction.; QTc, heart-rate corrected QT interval.

**Table S6. Gene Ontology Enrichment analysis of the mis-spliced genes in the conditional inducible QKI KO hearts.**

| **GO term**  **“biological process”** | **# background** | **#**  **dataset** | **#**  **expected** | **Fold Enrichment** | **P value** |
| --- | --- | --- | --- | --- | --- |
| cardiac muscle hypertrophy | 32 | 13 | 2.26 | 5.76 | 3.30E-02 |
| sarcomere organization | 36 | 14 | 2.54 | 5.52 | 2.13E-02 |
| myofibril assembly | 56 | 17 | 3.95 | 4.31 | 3.06E-02 |
| striated muscle cell development | 56 | 17 | 3.95 | 4.31 | 3.06E-02 |
| cardiac muscle cell development | 65 | 19 | ­4.58 | 4.15 | 1.33E-02 |
| cardiac cell development | 71 | 19 | 5 | 3.8 | 3.98E-02 |
| **GO term**  **“cellular component”** | **# background** | **#**  **dataset** | **#**  **expected** | **Fold Enrichment** | **P value** |
| cell cortex region | 36 | 13 | 2.54 | 5.12 | 1.58E-02 |
| A band | 34 | 12 | 2.40 | 5.01 | 4.12E-02 |
| Z disc | 108 | 38 | 7.61 | 4.99 | 1.49E-10 |
| I band | 118 | 40 | 8.32 | 4.81 | 9.05E-11 |
| calcium channel complex | 40 | 13 | 2.82 | 4.61 | 3.96E-02 |
| sarcomere | 169 | 51 | 11.91 | 4.28 | 1.59E-12 |
| **GO term**  **“molecular function”** | **# background** | **#**  **dataset** | **#**  **expected** | **Fold Enrichment** | **P value** |
| calmodulin binding | 146 | 33 | 10.29 | 3.21 | 1.49E-04 |
| actin filament binding | 186 | 35 | 13.11 | 2.67 | 3.39E-03 |
| actin binding | 376 | 70 | 26.50 | 2.64 | 2.07E-08 |
| cytoskeletal protein binding | 854 | 143 | 60.18 | 2.38 | 3.85E-16 |
| protein binding | 7009 | 566 | 493.95 | 1.15 | 1.49E-02 |
| DNA binding | 1729 | 77 | 121.85 | 0.63 | 2.38E-02 |

Gene ontology enrichment analysis was performed with the online tool PANTHER^2^ comparing the mis-spliced genes in ciQKI KO samples (expression ≥ 0.5 TPM; exon bins with absolute log2FC > 1, p-adj < 0.05) against all genes expressed in the ciQKI WT (≥ 0.5 TPM). #, number of genes. Fisher's Exact with Bonferroni correction for multiple testing.

**Supplemental references**

1. Bolger, A. M., Lohse, M. & Usadel, B. Trimmomatic: a flexible trimmer for Illumina sequence data. *Bioinformatics* **30**, 2114–2120 (2014).

2. Mi, H. *et al.* PANTHER version 16: a revised family classification, tree-based classification tool, enhancer regions and extensive API. *Nucleic Acids Res.* **49**, D394–D403 (2021).

3. Aufiero, S. *circRNAprofiler: circRNAprofiler: An R-Based Computational Framework for the Downstream Analysis of Circular RNAs*. (Bioconductor version: Development (3.11), 2020). doi:10.18129/B9.bioc.circRNAprofiler.

4. Haan, A. D. den *et al.* Organ Explant Culture of Neonatal Rat Ventricles: A New Model to Study Gene and Cell Therapy. *PLOS ONE* **8**, e59290 (2013).

**Unedited western blots shown in figures 2, 7 and S7.**


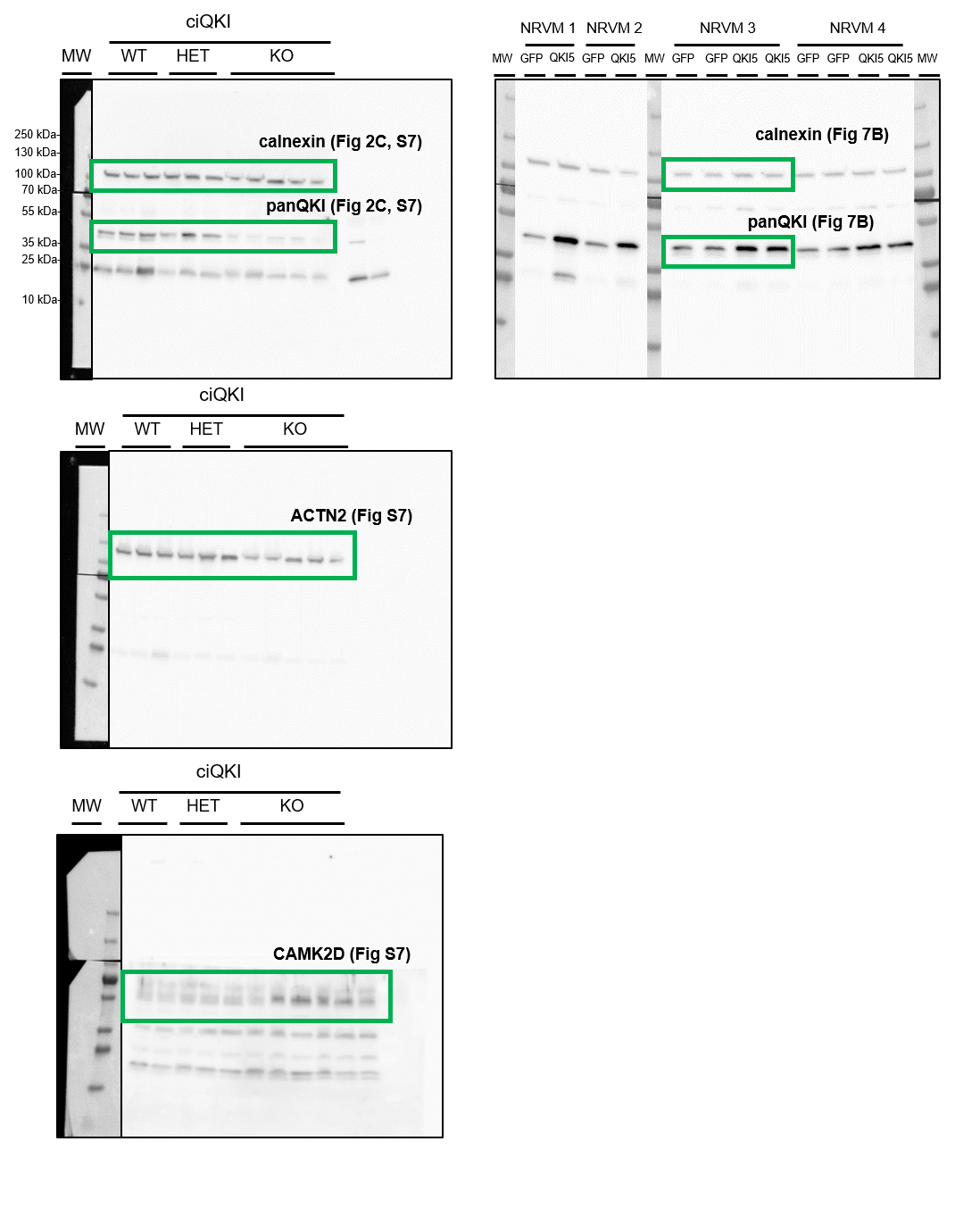


**Unedited gels from RT-PCRs shown in figure 4E.**


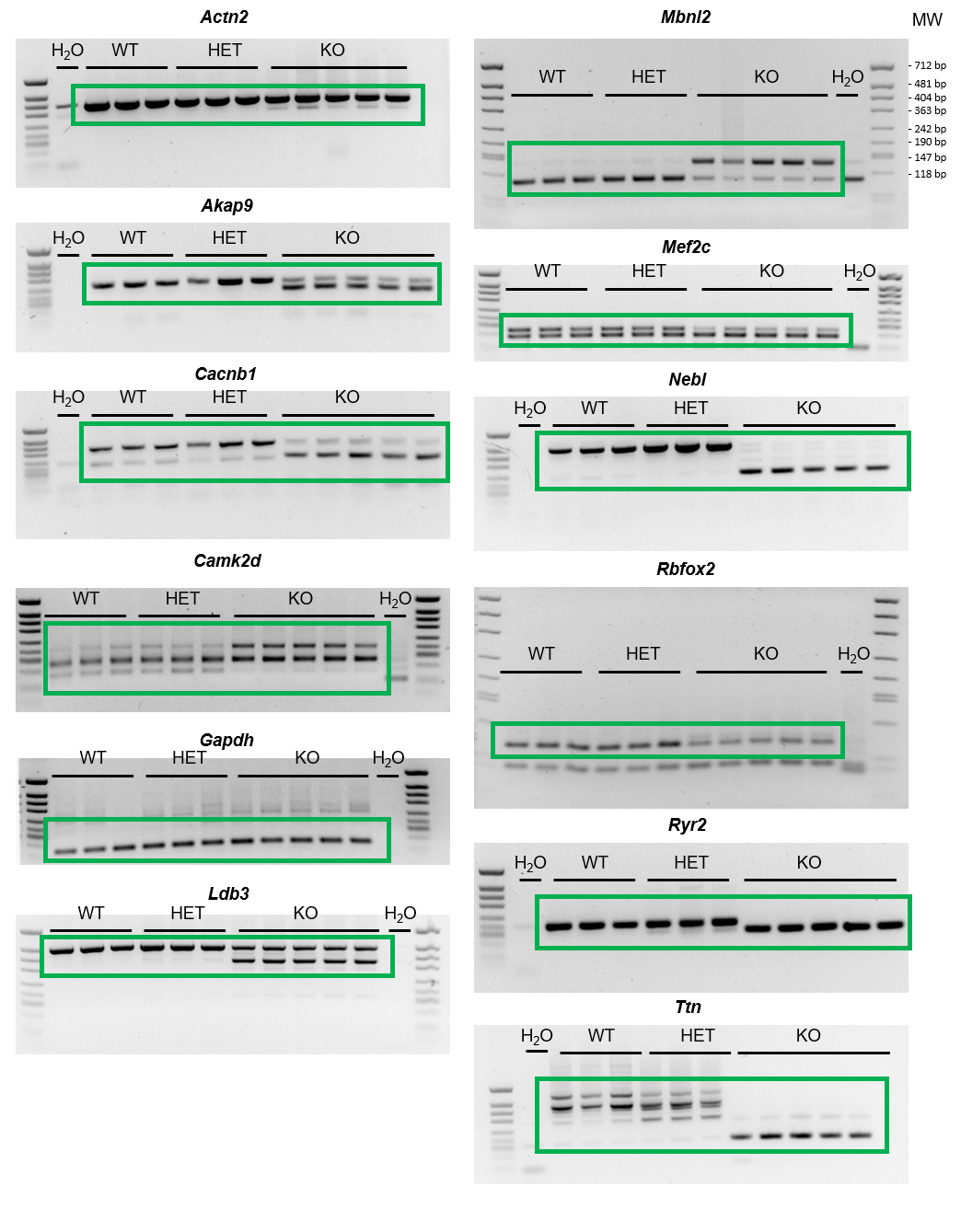


**Unedited gels from RT-PCRs shown in figure 6A (left column) and figure 7C (right column).**


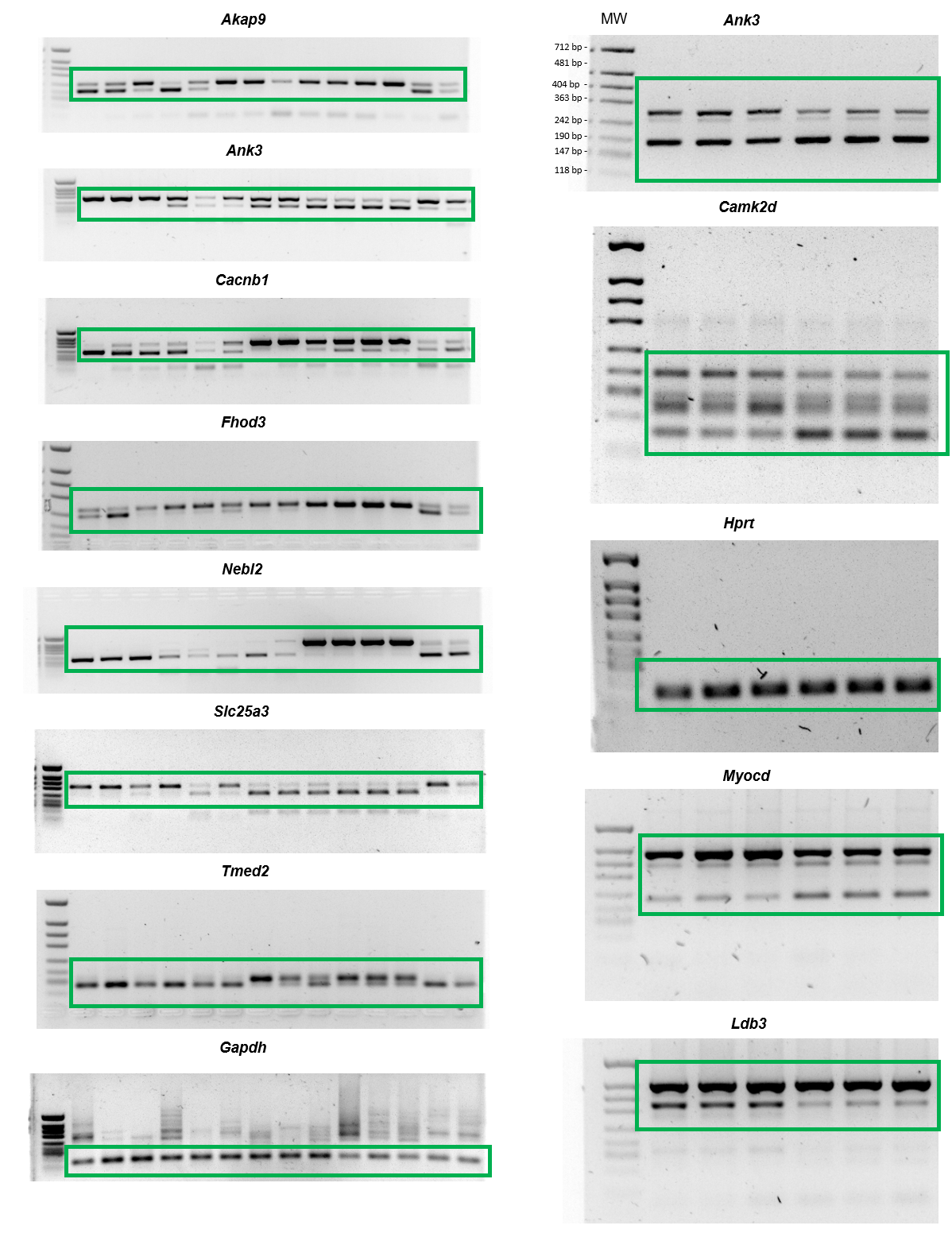

Supplement: cvad007_Supplementary_Data [file cvad007_supplementary_data.zip › CVR-2022-0586R2 - Supplemental methods.docx]
