# Supplementary figures and images for: The RNA-binding protein QKI governs a muscle-specific alternative splicing program that shapes the contractile function of cardiomyocytes
Source: Cardiovasc Res. 2023 Jan 11;119(5):1161–74. doi: 10.1093/cvr/cvad007 (PMC10202634; doi:10.1093/cvr/cvad007)

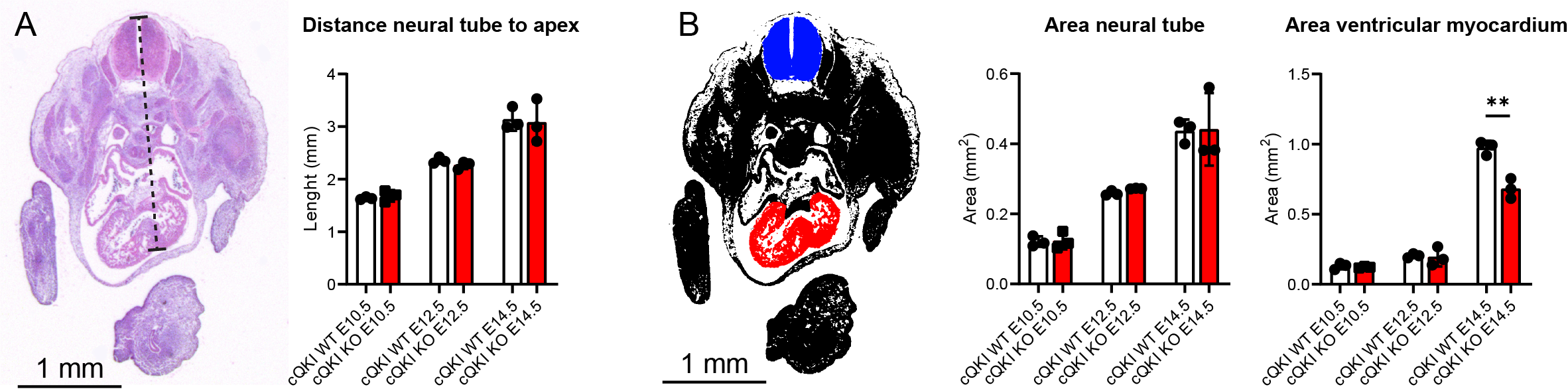

Supplement: cvad007_Supplementary_Data [file cvad007_supplementary_data.zip › Figure S1.tif]

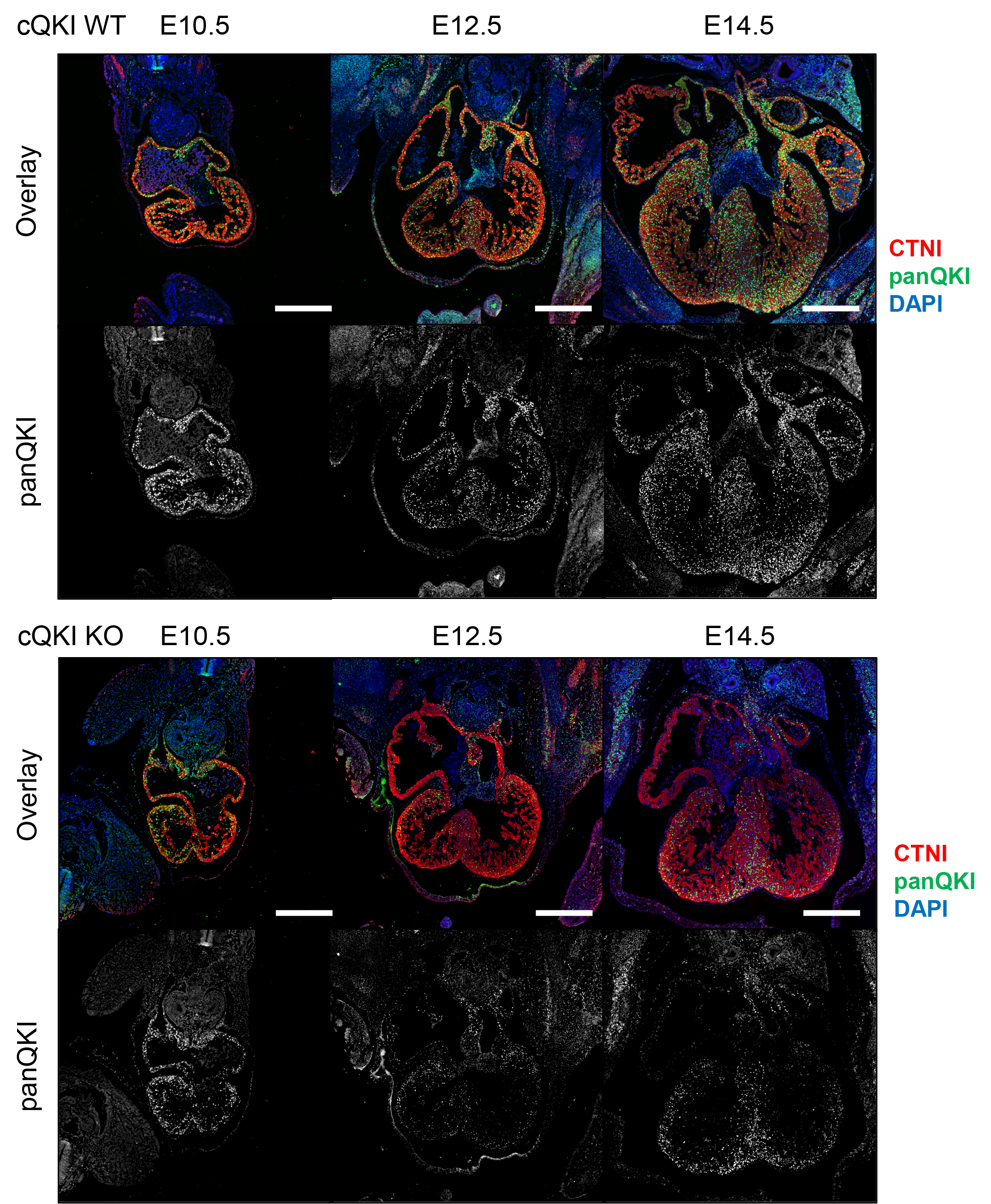

Supplement: cvad007_Supplementary_Data [file cvad007_supplementary_data.zip › Figure S2.tif]

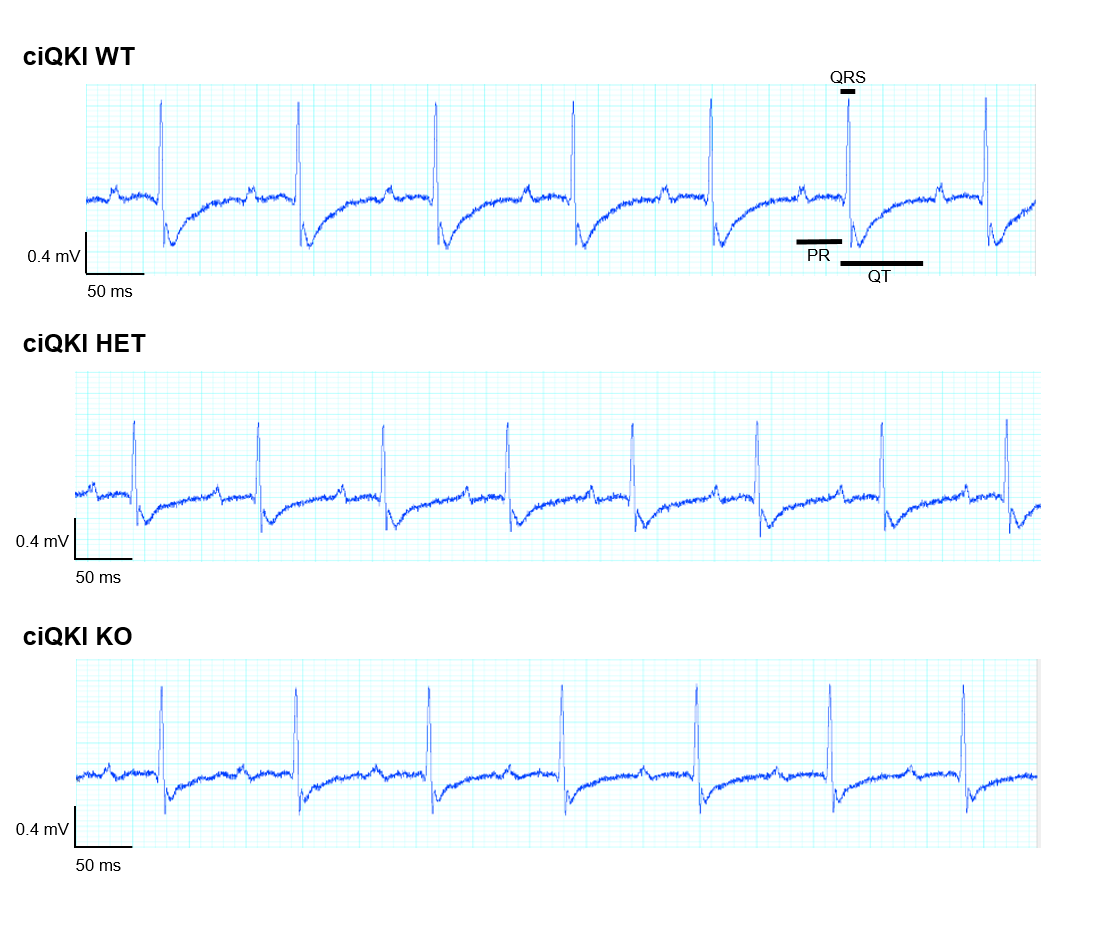

Supplement: cvad007_Supplementary_Data [file cvad007_supplementary_data.zip › Figure S3.tif]

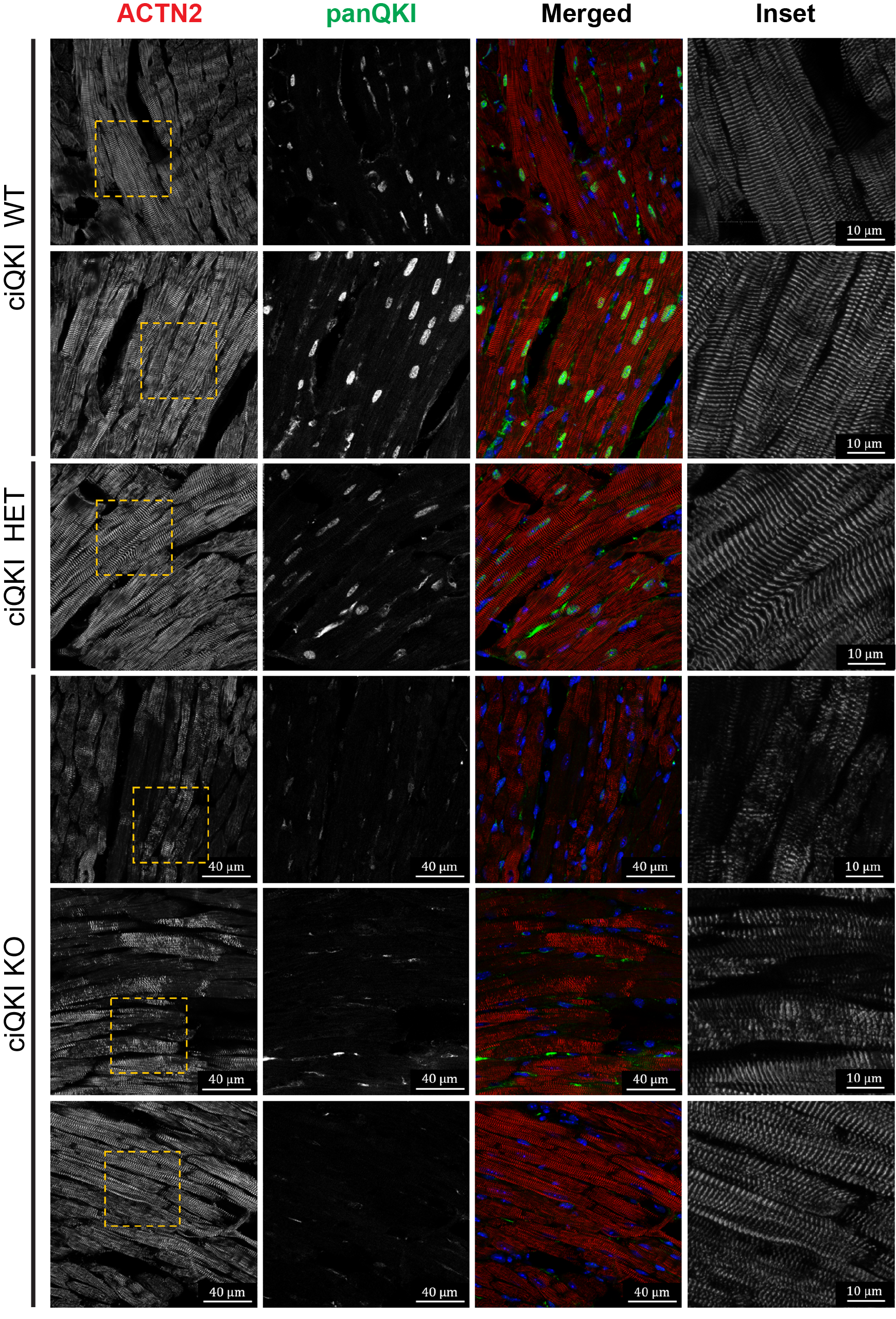

Supplement: cvad007_Supplementary_Data [file cvad007_supplementary_data.zip › Figure S4.tif]

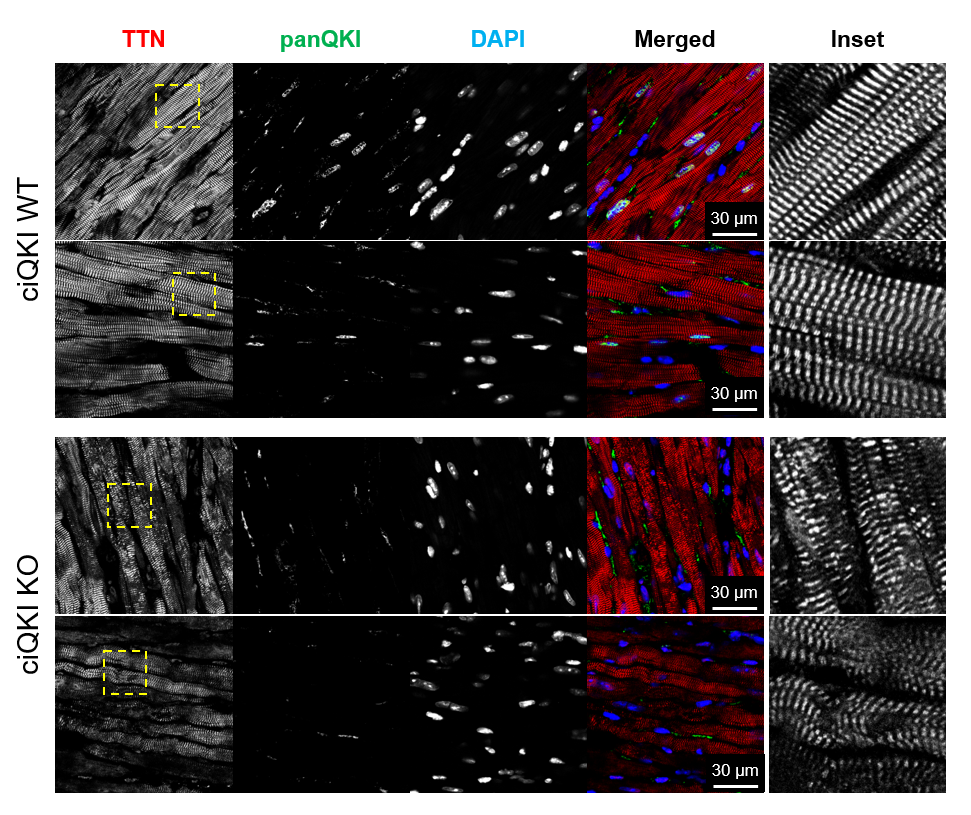

Supplement: cvad007_Supplementary_Data [file cvad007_supplementary_data.zip › Figure S5.tif]

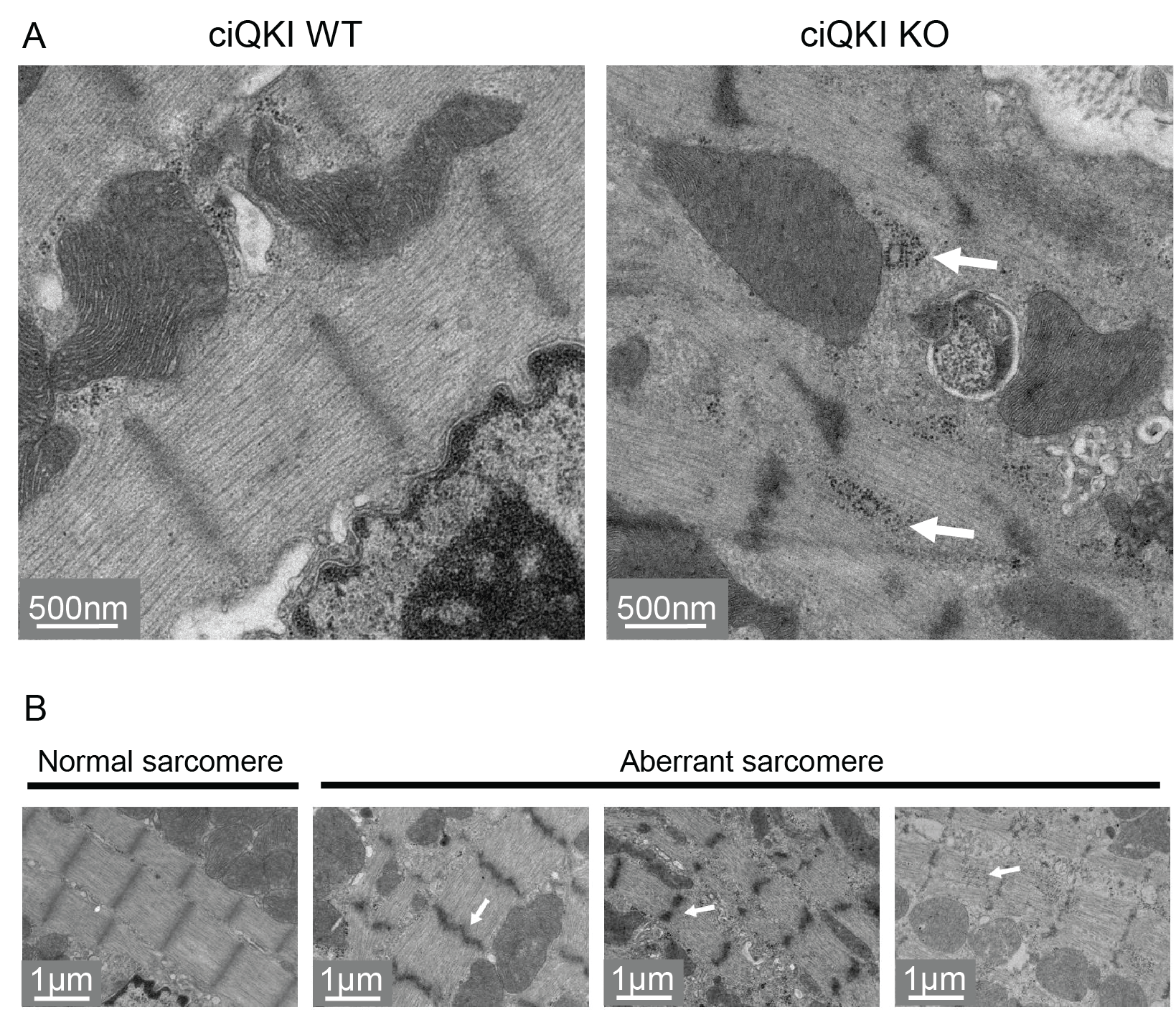

Supplement: cvad007_Supplementary_Data [file cvad007_supplementary_data.zip › Figure S6.tif]

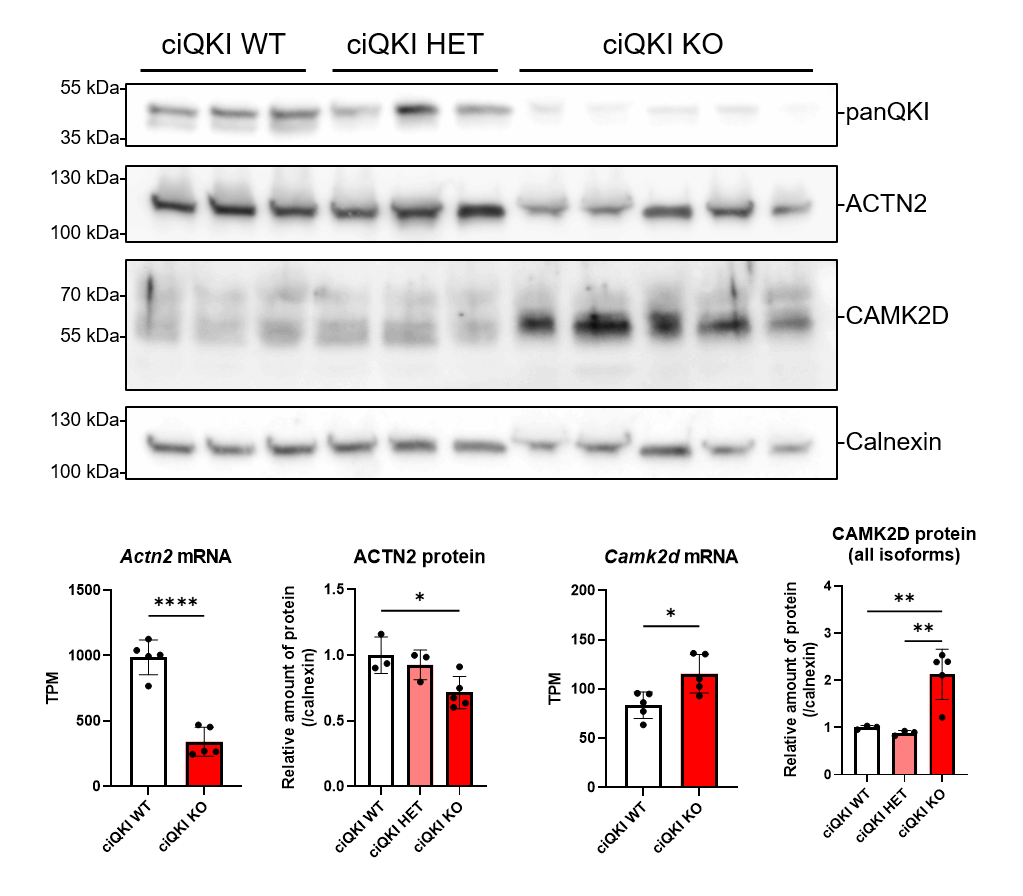

Supplement: cvad007_Supplementary_Data [file cvad007_supplementary_data.zip › Figure S7.tif]

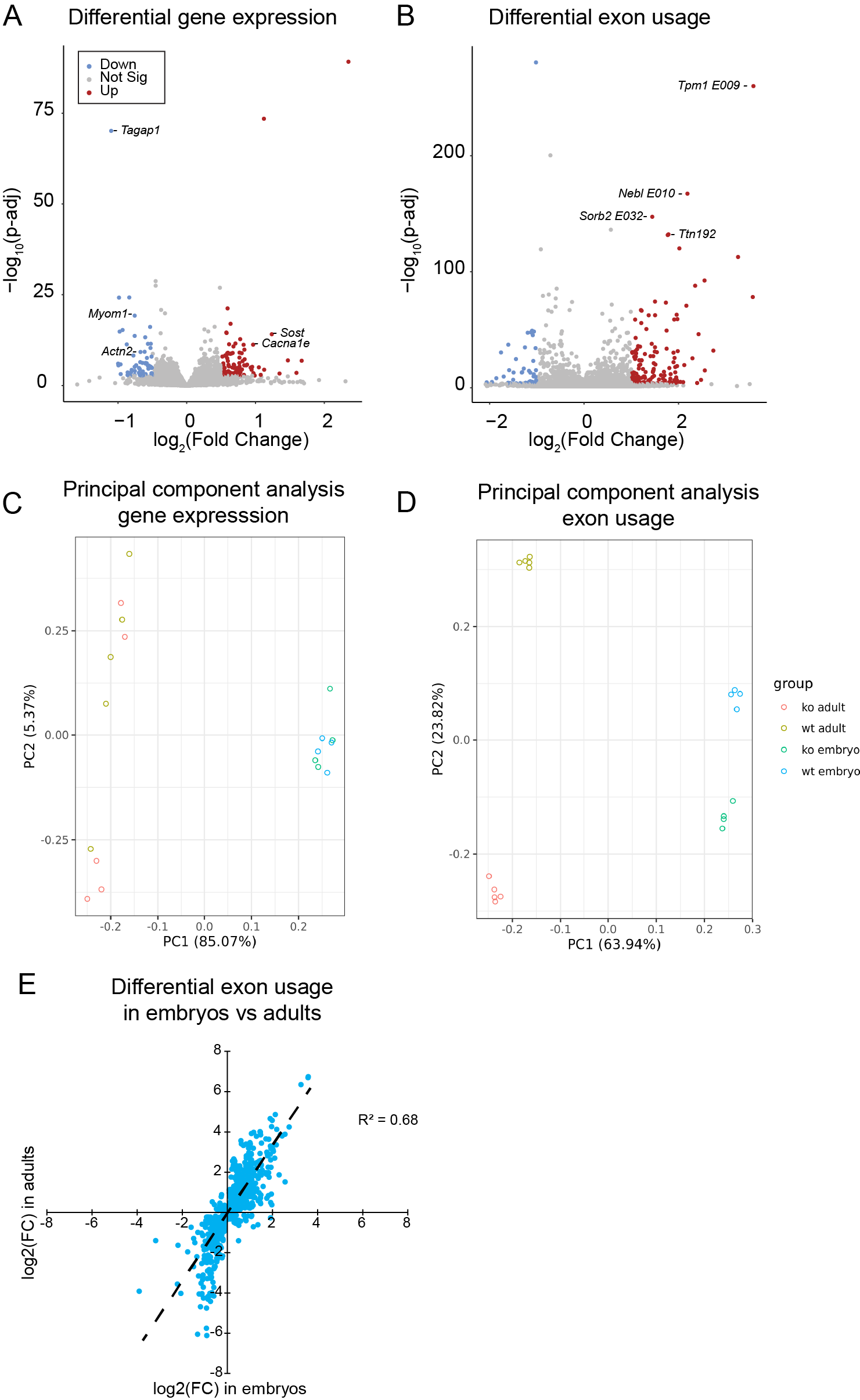

Supplement: cvad007_Supplementary_Data [file cvad007_supplementary_data.zip › Figure S8.tif]

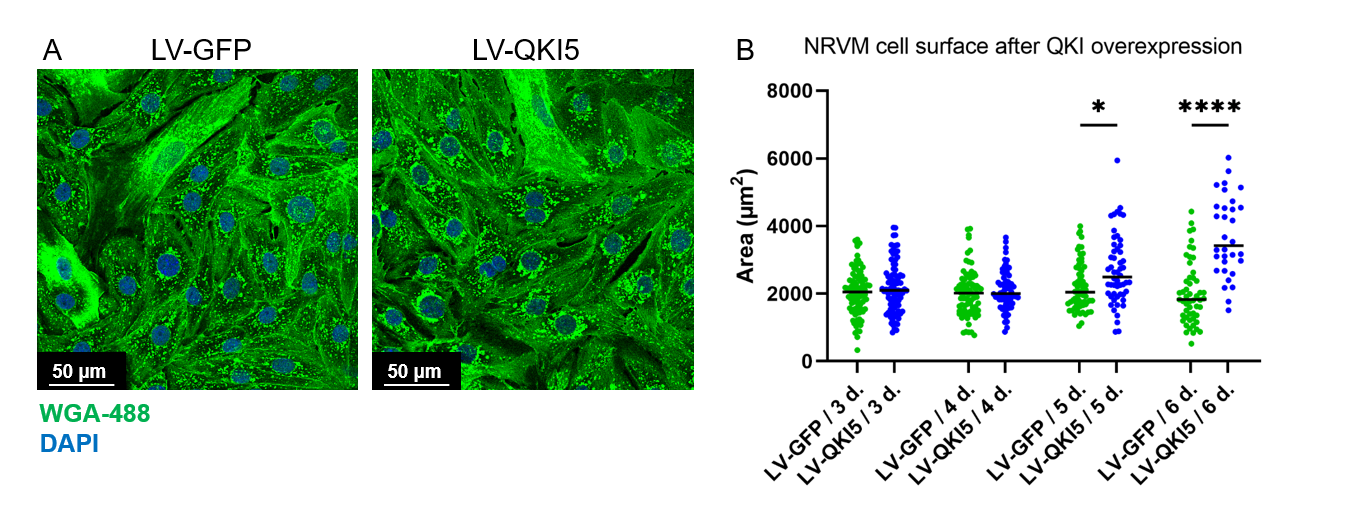

Supplement: cvad007_Supplementary_Data [file cvad007_supplementary_data.zip › Figure S9.tif]
